# Synthesis of DNA Building Blocks With Thiazole Fluorophores for Fluorescent Readout by Excimers and Energy Transfer

**DOI:** 10.1002/cplu.70263

**Published:** 2026-10-25

**Authors:** Jan Kunzmann, Anna‐Lena Ruopp, Hans‐Achim Wagenknecht

**Affiliations:** ^1^ Institute of Organic Chemistry Karlsruhe Institute of Technology (KIT) Karlsruhe Germany

**Keywords:** aminonaphthalimide, cyanovinylene, DNA, excimers, fluorescence, thiazole orange, UV/Vis absorption

## Abstract

Two new charged DNA building blocks were synthesized with thiazole orange (TO) and dibenzothiazole (DB) as fluorescent DNA base surrogates. They promote excimer formation and energy transfer within DNA architectures, because they were incorporated as fluorophore pairs into oligonucleotides such that they interact in an interstrand and intrastrand orientation upon hybridization. Both excimer formation and energy transfer result not only in fluorescence intensity changes, but also in pronounced redshifted emission. In addition, DB was investigated as an energy donor with TO as well as cyanovinylenes and aminonaphthalimides as energy acceptors in DNA. These combinations render DB as a versatile fluorophore for DNA detection and imaging. Such sensitive fluorescent probes basically enable sequence‐specific detection of nucleic acids not only through changes in fluorescence intensity but also by emission color as readout.

## Graphical abstract

Two new charged DNA building blocks were synthesized with thiazole orange (TO) and dibenzothiazole (DB) as fluorescent DNA base surrogates. They promote excimer formation and energy transfer within DNA architectures for fluorescent readout.

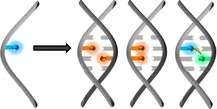

## 1 Introduction

Fluorescent nucleic acids comprise a central class of tools for molecular diagnostics, bioanalytical sensing, and live‐cell imaging. Sequence‐specific detection of DNA or RNA typically relies on hybridization‐sensitive probes with emission properties responding to changes in their local environment [1, 2]. While many such probes are based on fluorescence intensity modulation, spectral shifts of emission maxima provide a more robust readout, as they are less susceptible to artifacts arising from unspecific binding or fluctuations in probe concentration [3]. Pronounced emission shifts can be achieved through the formation of excited‐state complexes between neighboring chromophores embedded in DNA. Excimers are formed by two identical fluorophores and show a distinct redshifted emission without a fine structure, whereas exciplexes originate from interactions between two different dyes [4, 5, 6, 7]. Beyond these bichromophore architectures, multichromophore‐DNA architectures have been widely exploited to mediate Förster resonance energy transfer (FRET) [8, 9], enabling fluorescence‐based detection of structural features such as mismatches, mutations or modifications in DNA conformations [2, 10, 11, 12].

Among classic excimer‐forming dyes, pyrene has been extensively studied and successfully integrated into DNA [7, 13, 14, 15]. However, its practical application in diagnostics and imaging is limited by excitation in the ultraviolet region, which may induce DNA damage, as well as by pronounced sensitivity toward oxygen [16, 17]. Perylene bisimides also form excimers in duplex DNA with redshifted emission, but their fluorescence is efficiently quenched in the presence of guanine through photoinduced electron transfer [5, 18, 19]. Thiazole orange (TO) represents a widely used alternative, as it can be excited with visible light above 450 nm and exhibits a redox potential that precludes guanine oxidation. Consequently, TO has become a standard fluorophore for nucleic acid detection, for example, as SYBR Green I in PCR or as covalent dimer TOTO [4, 20, 21]. Several structural strategies have been followed to incorporate TO into nucleic acids in a hybridization‐sensitive manner. Seitz et al. introduced TO into peptide nucleic acids and later into DNA to generate “forced intercalation thiazole orange (FIT)” probes, which display pronounced fluorescence enhancement upon duplex formation and have been successfully applied for mRNA imaging [22, 23]. Alternative backbone modifications, such as acyclic D‐threoninol nucleic acids (aTNA), were reported by Asanuma et al. [24]. Furthermore, Okamoto et al. demonstrated that two adjacent TO chromophores placed next to each other in DNA can give rise to exciton‐coupled systems, termed “exciton‐controlled hybridization sensitive oligonucleotide” (ECHO) probes [25]. Time‐resolved experiments revealed that interstrand and interstrand TO dimers in DNA must be considered as mixtures of non‐fluorescent H aggregates, static, and dynamic excimers due to the conformational flexibility of double‐stranded DNA. The redshifted fluorescence of TO dimers in DNA mainly results from static excimers featuring the absorption of H aggregates [20]. Beyond TO‐based systems, cyanine dyes provide access to excimers, too. Chen et al. reported noncovalent intercalation of the cyanine dye K21 in DNA, where transient DNA breathing enables excimer formation accompanied by redshifted emission. Structurally, K21 has a dibenzothiazole (DB) core [26, 27].

In this work, we introduce two novel fluorescent DNA building blocks TO and DB, based on thiazole fluorophores. While TO serves as a benchmark excimer‐forming chromophore, DB, structurally derived from K21, was specifically designed for covalent incorporation into the DNA backbone, enabling precise positional control within defined DNA architectures (Figure 1). This design basically allows excimer formation in an interstrand orientation by placing two dyes into complementary DNA single strands, as well as in an intrastrand orientation by placing two dyes adjacent to each other in a single oligonucleotide. Importantly, DB further extends excimer‐based readout concepts by acting as an efficient energy donor in DNA duplexes. We therefore explore its ability to sensitize TO and the established fluorophores cyanovinylene and aminonaphthalimide, demonstrating how excimer formation and energy transfer can be used to create DNA architectures with a fluorescent readout.

**FIGURE 1:**
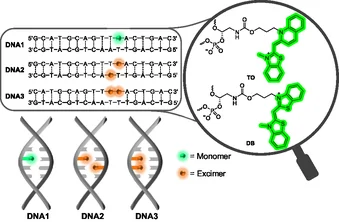
Structures of the fluorescent nucleotide analogs based on thiazole orange (TO) and dibenzothiazole (DB), DNA sequences **DNA1**–**DNA3** and schematic representation of the relative orientation of the fluorophores in these DNA double strands for excimer formation.

## 2 Results and Discussion

### 2.1 Synthesis of TO and DB DNA Building Blocks

TO was incorporated into oligonucleotides using (*S*)‐3‐amino‐1,2‐propandiol as an acyclic linker between the phosphodiester bridges. This chiral propanediol was selected due to its high stability and conformational flexibility compared to conventional 2‐deoxyfuranoside, allowing TO to be positioned as a nucleobase surrogate within the DNA [28, 29]. The presented building block was designed as a regioisomer of a former TO derivative [4]. The acyclic linker was attached through the quinoline moiety rather than the benzothiazole moiety. This altered attachment provides an alternative orientation of TO in the DNA for a potential fluorescent readout. For the synthesis of phosphoramidite **7** (Figure 2), 3‐chloropropan‐1‐ol was first converted into the more reactive 3‑iodopropan‐1‐ol (**1**) via the Finkelstein reaction [30]. Freshly prepared iodide **1** was subsequently reacted with 2‐methylquinoline, resulting in attachment of the propanol linker to the quinoline nitrogen via nucleophilic substitution to afford **2** in 42% yield [31]. To synthesize the modified benzothiazole **3**, 2‐methylthiobenzothiazole was *N*‐methylated using methyl iodide [32]. Condensation of modified quinoline **2** with benzothiazole **3** in basic conditions afforded the TO derivative **4** in 25% yield [33]. Activation of **4** with 1,1’‐carbonyldiimidazole (CDI) enabled subsequent coupling to linker **5**, yielding the carbamate‐linked intermediate **6** by 32% [33]. Linker **5** already bearing the DMT protecting group required for oligonucleotide synthesis was prepared according to the literature [28]. To incorporate the TO DNA building block into oligonucleotides, **6** was finally converted into phosphoramidite **7**.

**FIGURE 2:**
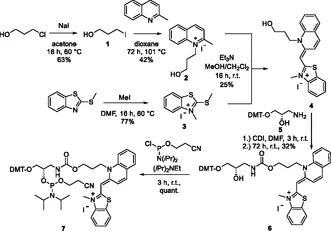
Synthesis of phosphoramidite **7** as building block for modification of DNA with TO.

To enable incorporation of DB as a fluorescent base surrogate into DNA, phosphoramidite **11** was synthesized using the same acyclic linker as described above (Figure 3). Compounds **1** and **3** were prepared according to the previously reported procedures. Nucleophilic substitution of compound **1** with 2‐methylbenzothiazole afforded the charged benzothiazole derivative **8** in 44% yield [33]. Subsequent base‐catalyzed condensation of **3** and **8** yielded DB **9** by 75%. Activation of **9** by CDI, followed by carbamate formation with linker **5**, provided intermediate **10** in 32% yield [33]. Finally, phosphoramidite formation of **10** furnished **11** suitable for covalent incorporation into oligonucleotides by solid‐phase DNA synthesis.

**FIGURE 3:**
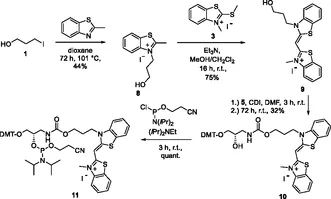
Synthesis of phosphoramidite **11** as a building block for modification of DNA with DB.

### 2.2 Fluorescent Dimers of TO in DNA

Phosphoramidite **7** was incorporated as a single modification into two oligonucleotides, single‐stranded (ss) **TO‐DNA1ss** and corresponding TO‐modified counterstrand using a slightly modified automated solid‐phase DNA synthesis protocol (see Supporting Information). Both strands contain one TO modification but differ in their sequences to ensure mutual complementary. Upon annealing, **TO‐DNA2** carries two TO fluorophores positioned so close to each other in the sequence to basically form an interstrand dimer. In contrast, **TO‐DNA1** contains only a single TO modification in the duplex because **TO‐DNA1ss** was annealed with the unmodified counterstrand. Single‐stranded **TO‐DNA1ss** exhibits a distinct absorption band in the visible region with a maximum at *λ* = 490 nm and a shoulder at *λ* = 470 nm, the characteristic fine structure of this chromophore, but hypsochromically shifted by approximately 20 nm due to interactions in DNA (Figure 4). Double‐stranded **TO‐DNA1**, bearing only a single TO chromophore, shows a similar absorption profile with two local maxima at *λ* = 470 nm and *λ* = 490 nm, indicating that the UV/vis absorption properties are not significantly affected by duplex formation. In line with the designed interstrand dye–dye interaction, the absorption spectrum of **TO‐DNA2** markedly differs from the single modified oligonucleotides. Notably, the relative intensities of the two absorption maxima are inverted by a maximum at *λ* = 470 nm and a shoulder at *λ* = 490 nm. This inversion of the absorption fine structure is indicative of excitonic coupling between the two antiparallel‐oriented TO chromophores [20]. Fluorescence emission spectra were recorded upon excitation at *λ* = 490 nm. The modified **TO‐DNA1ss** and the double‐strand **TO‐DNA1** exhibit a fluorescence maximum at *λ* = 553 nm with a shoulder at *λ* = 530 nm, indicating that duplex formation does not significantly affect the emission properties of the chromophore as a single modification in DNA; only the quantum yield is increased from *Φ*_F_ = 0.040 for **TO‐DNA1ss** to *Φ*_F_ = 0.077 for the double strand (Table 1). Literature reports on the TO monomer incorporated into DNA to display an emission maximum at *λ* = 530 nm with a shoulder at *λ* = 565 nm [4, 20]. The slightly shifted fluorescence for the TO chromophore in **TO‐DNA1** must therefore be attributed to the different interactions of the chromophore within the DNA base stack due to the chosen acyclic linker between the phosphodiester bridges. In contrast, the emission of **TO‐DNA2** differs markedly and exhibits a pronounced bathochromic shift with a fluorescence maximum at *λ* = 575 nm. Simultaneously, the characteristic fine structure of TO emission is lost, resulting in a broad, structureless emission band. In addition, a significant drop in the fluorescence quantum yield is observed; **TO‐DNA1** shows nearly twice the quantum yield compared to **TO‐DNA2** (*Φ*_F_ = 0.044). Taken together, these results provide compelling evidence for the formation of an interstrand TO dimer in **TO‐DNA2,** resulting in an emission from a static excimer. The bathochromic emission shift is sufficiently pronounced to be readily observed by the naked eye upon excitation with a handheld UV lamp (*λ* = 254 nm). While the TO monomer (**TO‐DNA1**) emits green fluorescence, the TO dimer (**TO‐DNA2**) exhibits orange emission, typical for the TO excimer. The determined melting temperatures (*T*_m_) support the formation of a static dimer in **TO‐DNA2** because chromophores as single base surrogates in DNA typically drop the melting temperatures of DNA by a few °C. The melting temperatures of **TO‐DNA1** and **TO‐DNA2**, however, are very similar (*T*_m_ = 66.9 and 66.2 °C, respectively), although the latter double strand was modified by two chromophores, indicating that chromophore interactions in the TO dimer stabilize **TO‐DNA2**.

**FIGURE 4:**
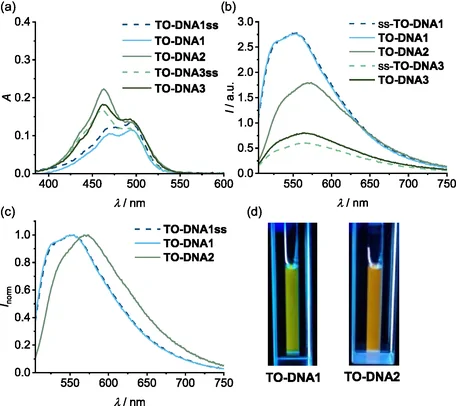
(a) UV/Vis absorption; (b) fluorescence of **TO‐DNA1ss**, **TO‐DNA1**, **TO‐DNA2**, **TO‐DNA3ss,** and **TO‐DNA3** (*λ*_exc_ = 490 nm, 2.5 μM in 10 mM Na‐P_i_ buffer, 250 mM NaCl, pH 7); (c) normalized fluorescence of **TO‐DNA1ss**, **TO‐DNA1**, **TO‐DNA2** (2.5 μM in 10 mM Na‐P_i_ buffer, 250 mM NaCl, pH 7); and (d) images of cuvettes filled with **TO‐DNA1**, **TO‐DNA2** (2.5 μM in 10 mM Na‐P_i_ buffer, 250 mM NaCl, pH 7) excited by handheld UV lamp (254 nm).

**TABLE 1:** Quantum yield (*Φ*_F_), melting temperatures (*T*_m_), maximum of the absorbance (*λ*_abs_), and fluorescence (*λ*_em_) of single strands **TO‐DNA1ss** and **TO‐DNA3ss** and double strands **TO‐DNA1**, **TO‐DNA2**, and **TO‐DNA3** (*λ*_exc_ = 490 nm, 2.5 μM in 10 mM Na‐P_i_ buffer, 250 mM NaCl, pH 7).

DNA	*Φ*_F_	*T*_m_, °C	*λ*_abs_, nm	*λ*_em_, nm
**TO‐DNA1ss**	0.040	—	472/494	553
**TO‐DNA3ss**	0.020	—	462/492	565
**TO‐DNA1**	0.077	66.9	470/494	553
**TO‐DNA2**	0.044	66.2	462/495	575
**TO‐DNA3**	0.021	61.0	462/493	565

To assess the importance of chromophore geometry within DNA for dimer formation, the parallel intrastrand configuration was investigated. Two TO monomers were positioned adjacently to each other within the same strand in **TO‐DNA3ss**. Both **TO‐DNA3ss** and the double‐strand **TO‐DNA3** (after annealing with the complementary unmodified counterstrand) exhibit an absorption maximum at *λ* = 462 nm with a shoulder at *λ* = 495 nm. As observed for the interstrand TO dimer, the hypsochromically shifted shoulder represents the dominant absorption, again indicating the formation of a static dimer. Duplex formation does not significantly influence the absorption properties of the intrastrand TO dimer. Upon excitation at *λ* = 490 nm, both **TO‐DNA3ss** and the double‐strand **TO‐DNA3** display a bathochromically shifted emission at *λ* = 565 nm, characteristic of TO dimer emission. Similar to the absorption, hybridization does not affect dye–dye interactions in the intrastrand arrangement, demonstrating that the DNA architecture of the single strand is sufficient to induce dimer formation with redshifted fluorescence. Notably, in comparison to the interstrand TO dimer (**TO‐DNA2**), the intrastrand dimer (**TO‐DNA3**) exhibits a reduced fluorescence quantum yield of *Φ*_F_ = 0.021. This pronounced decrease indicates that the parallel arrangement of two adjacent TO chromophores within the same strand promotes additional nonradiative pathways, probably by H aggregates, resulting in more efficient fluorescence quenching [20]. Overall, the present TO building block designed as a regioisomer to a former TO derivative [4] does not show improved fluorescent readout. Attaching the acyclic linker to the quinoline alters the fluorescence properties in dimers. However, the redshift of TO excimers is more pronounced for the former regioisomer where the linker was attached to the benzothiazole. These differences indicate that the attachment site is critical for the chromophore orientation and the geometry of dimers in DNA.

### 2.3 Fluorescent Dimers of DB in DNA

For the investigation of interstrand DB dimers, phosphoramidite **11** was incorporated into **DB‐DNA1ss** and the corresponding complementary strand (for **DB‐DNA2**) using the modified automated solid‐phase DNA synthesis protocol mentioned above for TO (see the Supporting Information). **DB–DNA1ss** as well as the double‐strand **DB‐DNA1** containing a single DB modification exhibit an absorption maximum at *λ* = 430 nm with a shoulder at *λ* = 407 nm (Figure 5). As previously observed for TO, the fine structure is retained, with the hypsochromically shifted band representing the dominant absorption feature for the monomeric DB chromophore. Duplex formation does not significantly affect the absorption properties of DB when incorporated into DNA as a single modification. In contrast, an inversion of the absorption fine structure is observed when two DB fluorophores are positioned antiparallel in **DB‐DNA2**. In this case, the absorption displays a maximum at *λ* = 407 nm, with a shoulder at *λ* = 430 nm. The altered absorption pattern observed for **DB‐DNA2** reflects electronic interactions between two antiparallel DB chromophores and supports the formation of static dimers in this interstrand DB dimer.

**FIGURE 5:**
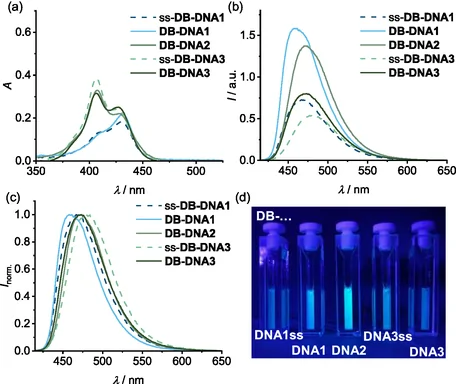
(a) UV/Vis absorption; (b) fluorescence of **DB‐DNA1ss**, **DB‐DNA1**, **DB‐DNA2**, **DB‐DNA3ss,** and **DB‐DNA3** (*λ*_exc_ = 400 nm, 2.5 μM in 10 mM Na‐P_i_ buffer, 250 mM NaCl, pH 7); (c) normalized fluorescence of **DB‐DNA1ss**, **DB‐DNA1**, **DB‐DNA2** (*λ*_exc_ = 400 nm, 2.5 μM in 10 mM Na‐P_i_ buffer, 250 mM NaCl, pH 7); and (d) cuvettes of DB‐DNA samples (2.5 μM in 10 mM Na‐P_i_ buffer, 250 mM NaCl, pH 7) excited by handheld UV lamp (360 nm).

To investigate the fluorescence properties of DB‐modified oligonucleotides, **DB‐DNA1ss**, **DB‐DNA1,** and **DB‐DNA2** were excited at *λ* = 400 nm. Comparison of the single strand **DB‐DNA1ss** with its double strand **DB‐DNA1** reveals a pronounced influence of duplex formation on the emission behavior of DB. Upon hybridization, the fluorescence intensity increases significantly, which is also reflected in the fluorescence quantum yield. The quantum yield increases from 33.3% for **DB‐DNA1ss** to 66.5% for **DB‐DNA1** (Table 2). In addition to this intensity enhancement, a shift in the emission maximum is observed. While **DB‐DNA1ss** displays a slightly redshifted maximum at *λ* = 466 nm, the double‐strand **DB‐DNA1** shows an emission maximum at *λ* = 460 nm. This shift of 6 nm indicates that DB in the single strand can interact with a second dye in another single strand by interstrand assembly. In contrast, within the closed double‐helical structure of DNA, the chromophore is embedded in the base stack and remains spatially isolated from other DB units. When two DB chromophores are positioned within **DB‐DNA2**, the interstrand interactions are enforced. As a result, the emission maximum is shifted to *λ* = 472 nm, corresponding to a bathochromic shift of 12 nm relative to that of **DB‐DNA1**. Simultaneously, the fluorescence quantum yield decreases markedly, with **DB‐DNA2** exhibiting a quantum yield of 30.0%, which is comparable to that of single‐stranded **DB‐DNA1ss**. This behavior is consistent with the formation of static DB excimers in **DB‐DNA2**, giving rise to redshifted emission accompanied by increased nonradiative decay pathways. This is supported by the melting temperatures: **DB‐DNA2** shows a slightly higher *T*_m_ than **DB‐DNA1** (*T*_m_ = 68.0 and. 66.0 °C, respectively), indicating a stabilizing effect by chromophore interactions in the DB dimer **DB‐DNA2**.

**TABLE 2:** Quantum yield (*Φ*_F_), melting temperatures (*T*_m_), maximum of absorbance (*λ*_abs_), fluorescence (*λ*_em_) of the single strands **DB‐DNA1ss**, **DB‐DNA3ss**, and the duplexes **DB‐DNA1**, **DB‐DNA2** and **DB‐DNA3**.

DNA	*Φ*_F_	*T*_m_, °C	*λ*_abs_, nm	*λ*_em_, nm
**DB‐DNA1ss**	0.333	—	407/430	466
**DB‐DNA3ss**	0.153	—	407/428	487
**DB‐DNA1**	0.665	66.0	407/430	460
**DB‐DNA2**	0.300	68.0	407/430	472
**DB‐DNA3**	0.221	55.0	407/428	480

In addition to the discussed interstrand arrangement, intrastrand DB dimers were investigated by incorporating adjacent chromophores into the single‐strand **DB‐DNA3ss**. The absorption of **DB‐DNA3ss** and its double‐strand **DB‐DNA3** are identical and closely mirror those observed for the interstrand DB dimer in **DB‐DNA2**. The hypsochromically shifted band at *λ* = 407 nm constitutes the dominant absorption feature, accompanied by a shoulder at *λ* = 430 nm. This characteristic absorption fine structure is consistent with the formation of static dimers. Upon excitation at *λ* = 400 nm, **DB‐DNA3ss** and **DB–DNA3** differ. **DB–DNA3ss** exhibits an emission maximum at *λ* = 487 nm, whereas **DB–DNA3** shows a slightly blueshifted maximum at *λ* = 480 nm. Notably, both intrastrand systems display bathochromically shifted emission relative to the single‐modified strands (**DB–DNA1ss** and **DB–DNA1**) and even compared to the interstrand DB dimer in **DB–DNA2**. The parallel arrangement of adjacent DB chromophores within a single strand therefore favors a more pronounced red shift of the emission. Together with the absorption, these findings also support the formation of static DB eximers assembly in intrastrand DNA architectures. As observed for TO, the intrastrand DB dimer exhibits markedly reduced fluorescence quantum yield. Quantum yields of 15.3% for **DB–DNA3ss** and 22.1% for **DB–DNA3** were determined, indicating that the parallel chromophore arrangement promotes additional nonradiative decay pathways and result in fluorescence quenching. Thus, DB reproduces the geometry‐dependent aggregate signatures observed for TO, while providing a distinct emission window and donor functionality.

### 2.4 DB‐Mixed Fluorescent Dimers in DNA

DB exhibits fluorescence in double‐stranded DNA at *λ* = 460 nm, which overlaps with the absorption of selected dyes acting as potential energy acceptors, including TO (*λ*_abs_ = 490 nm), cyanovinylenes CV1 (*λ*_abs_ = 500 nm), and CV2 (*λ*_abs_ = 515 nm), as well as aminonaphthalamides AN1 (*λ*_abs_ = 453 nm) and AN2 (*λ*_abs_ = 453 nm). The DNA building blocks CV1, CV2, AN1, and AN2 as well as the corresponding modified oligonucleotides were synthesized according to the literature [29]. To explore the energy transfer capabilities of DB as an energy donor with those selected other fluorophores, single‐strand **DB‐DNA1cs** was used (Figure 6) and annealed with complementary counterstrands **X‐DNA1ss** modified by the second chromophore to the double strands **X‐DNA4** with X = TO, CV1, CV2, AN1, and AN2.

**FIGURE 6:**
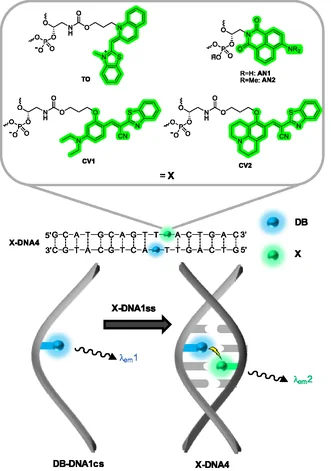
Structures of the fluorescent nucleotide analogs thiazole orange (TO), aminonaphthalimides (AN1 and AN2), and cyanovinylenes (CV1 and CV2) as energy acceptors in single‐stranded **X‐DNA1ss** annealed with **DB‐DNAss** to double‐strands **X‐DNA4** (with X = TO, CV1, CV2, AN1 or AN2) and schematic representation of the energy transfer from DB to these acceptor chromophores.

Upon annealing, antiparallel donor–acceptor pairs are positioned within the DNA duplexes, enabling energy transfer from excited DB as a donor to the respective acceptors. For **TO‐**, **CV1‐,** and **CV2‐DNA4**, the UV/Vis absorption shows the characteristic monomer absorption bands of DB and the respective acceptor dyes, whereas for **AN1‐** and **AN2‐DNA4,** the absorption of DB dominates due to spectral overlap (Figure 7). All duplexes of **X‐DNA4** were excited at *λ* = 400 nm, which primarily or even selectively excites DB. For comparison, the acceptor dyes were also excited at *λ* = 400 nm as a single modification in single strands **X‐DNA1ss** and corresponding double strands **X‐DNA1** (annealed by the complementary unmodified counterstrand). In these DNA samples, only weak fluorescence intensity is observed, confirming that the enhanced acceptor emission in the donor–acceptor duplexes **X‐DNA4** originates from energy transfer by excited DB. First, when **TO–DNA1ss** and **TO–DNA1** are excited at *λ* = 400 nm, only weak fluorescence at *λ* = 532 nm is detected. In contrast, **TO‐DNA4** displays both the DB donor emission at *λ* = 465 nm and the strongly enhanced TO acceptor emission at *λ* = 532 nm as a result of energy transfer. Second, comparable donor–acceptor behavior is observed for CV1 and CV2. They differ in their structural flexibility: CV1 bears two flexible ethyl substituents, and CV2 adopts a rigid, cyclized structure. When incorporated into the corresponding single strands **X‐DNA1ss** and excited at *λ* = 400 nm, both dyes display only weak fluorescence at their characteristic maxima (*λ*_em_ = 532 nm for CV1 and *λ*_em_ = 550 nm for CV2). Annealing with DB to double strands results in a pronounced change of emission. **CV1‐DNA4** exhibits emission of DB at *λ* = 465 nm together with a second and intense emission at *λ* = 552 nm by energy transfer to CV1. A similar response is observed for **CV2‐DNA4**, which displays both the DB emission and a redshifted acceptor fluorescence of CV2 at *λ* = 563 nm. Third, aminonaphthalimides AN1 and AN2 differ in their substitution at the the amino group: AN1 bears the protic amino group (NH_2_), whereas AN2 contains the dimethylamino substituent (NMe_2_). Upon excitation at *λ* = 400 nm, **AN1‐DNA1ss** and **AN1‐DNA1** exhibit weak fluorescence with a maximum at *λ* = 532 nm, similar to **AN2‐DNA1ss** and **AN2–DNA1** at *λ* = 525 nm. For **AN1‐DNA4** and **AN2‐DNA4**, emission of DB at *λ* = 465 nm occurs together with the overlapping acceptor emission bands of AN1 and AN2 as a result of energy transfer. Notably, for **TO‐DNA4** the donor and acceptor emission bands exhibit comparable intensities, whereas for **CV1‐DNA4**, **CV2‐DNA4**, **AN1‐DNA4,** and **AN2‐DNA4** the acceptor emission dominates. The energy transfer efficiencies were calculated according to *E* = 1 − *I*_D_/*I*_DA_ with the fluorescence intensity *I*_D_ of the donor DNA (**DB‐DNA1**) and the fluorescence intensity *I*_DA_ of the donor–acceptor DNA, both in the fluorescence range of DB. These energy‐transfer efficiencies range from 80% to 97%; the highest efficiency was observed for CV1 (97%), followed by CV2 (93%) and TO (92%), whereas AN1 and AN2 exhibited lower efficiencies of 83% and 80%, respectively. The quantum yields of these double‐strands range from 0.151 to 0.361 (Table 3). Taken together, DB acts as a versatile donor that sensitizes selected acceptors in a duplex‐defined geometry, expanding the fluorescence readout beyond excimer emission. As an acceptor, the best choices are CV1 and CV2 with well‐separated donor and acceptor emission ranges, high quantum yields (22% and 26%), high energy transfer efficiencies (97% and 93%), and fluorescence lifetimes (*τ*_2_ = 8.15 and 5.55 ns) suitable for fluorescent imaging purposes.

**FIGURE 7:**
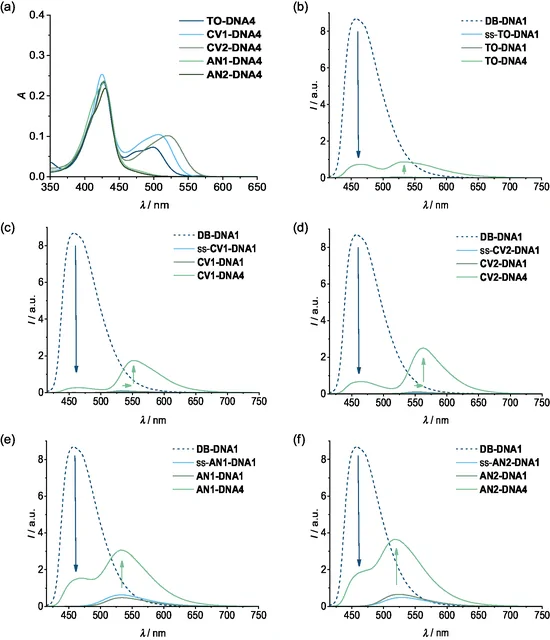
(a) UV/Vis absorption of **X‐DNA4**; (b) fluorescence of **TO‐DNA1ss**, **TO‐DNA1,** and **TO‐DNA4**; (c) **CV1‐DNA1ss**, **CV1‐DNA1,** and **CV1‐DNA4**; (d) fluorescence of **CV2‐DNA1ss**, **CV2‐DNA1,** and **CV2‐DNA4**; (e) fluorescence of **AN1‐DNA1ss**, **AN1‐DNA1,** and **AN‐DNA4**; and (f) fluorescence of **AN2‐DNA1ss**, **AN2‐DNA1,** and **AN2‐DNA4**; (*λ*_exc_ = 400 nm, 2.5 μM in 10 mM Na‐P_i_ buffer, 250 mM NaCl, pH 7).

**TABLE 3:** Quantum yield (*Φ*_F_), melting temperatures (*T*_m_), maximum of absorbance (*λ*_abs_), and fluorescence (*λ*_em_) of **TO‐DNA4**, **CV1‐DNA4**, **CV2‐DNA4**, **AN1‐DNA4**, and **AN2‐DNA4**.

DNA	*Φ*_F_	c_1_	*τ*_1_, ns	c_2_	*τ*_2_, ns
**TO‐DNA4**	0.151	0.51	1.05	0.49	3.58
**CV1‐DNA4**	0.217	0.73	3.66	0.27	8.15
**CV2‐DNA4**	0.263	0.58	2.71	0.42	5.55
**AN1‐DNA4**	0.361	0.05	1.48	0.95	9.97
**AN2‐DNA4**	0.080	0.16	2.48	0.84	13.3

## 3 Conclusion

In conclusion, we have developed two novel synthetic fluorescent DNA building blocks for TO and DB as thiazole‐derived fluorophores that enable excited‐state interactions in the dimeric form, both in the interstrand and intrastrand orientation in respective DNA architectures. The new TO nucleotide analog provides access to static inter‐ and intrastrand excimer formation as a fluorescence readout in DNA. Based on this structural concept, the DB nucleotide analog extends the fluorescent readout by static excimer formation and energy transfer donor. DB undergoes efficient energy transfer with a selected range of acceptor dyes including TO, CV1, CV2, AN1 and AN2. The excimer emission and energy transfer‐based fluorescence readouts significantly expand the toolbox of DNA‐based fluorescent probes and provide a versatile platform for advanced fluorescence detection and imaging of nucleic acids in general. The optic‐spectroscopic properties of TO‐, DB‐, and mixed DB‐dimers in DNA underscore the versatility of thiazole‐derived fluorophores as nucleic acid‐sensitive dyes and thereby expand the available toolbox of fluorescent probes for nucleic acids with turn‐on and redshift as fluorescent readouts.

## Funding

This work was supported by the Deutsche Forschungsgemeinschaft (Wa 1386/26‐1).

## Conflicts of Interest

The authors declare no conflict of interest.

## Supporting information

Supplementary Material

## Data Availability

All data supporting this study are available in the Supporting Information (SI).
