# Supplementary material for: Synthesis of DNA Building Blocks With Thiazole Fluorophores for Fluorescent Readout by Excimers and Energy Transfer
Source: Chempluschem. 2026 Oct 25;91(10):e70263. doi: 10.1002/cplu.70263 (PMC13696038; doi:10.1002/cplu.70263)
Supplement: Supplementary file 1 [file CPLU-91-e70263-s001.pdf]

Synthesis of DNA Building Blocks with Thiazole Fluorophores for Fluorescent Readout by Excimers and Energy Transfer

Jan Kunzmann,[a] Anna-Lena-Ruopp,[a] Hans-Achim Wagenknecht*[a]

[a]Institute of Organic Chemistry, Karlsruhe Institute of Technology (KIT),

Fritz-Haber-Weg 6, 76131 Karlsruhe, Germany

*Corresponding author, E-Mail: Wagenknecht@kit.edu

Supporting Information

Table of content

1. Materials and methods 1

2. Oligonucleotide synthesis 2

3. Synthesis of TO and DB building blocks 7 und 11 3

4. Images of NMR spectra 11

5. Images of MS analyses 18

6. Synthesis of Oligonucleotides 21

6.1 Images of HPLC analyses 21

6.2 Images of DNA MS analysis 24

7. Additional optical spectra of DB-mediated energy transfer 28

8. Synthesis protocol for modified DNA 29

1. Materials and methods

Reagents and solvents were obtained from *Sigma Aldrich*, *BLDpharm*, *Thermo Fisher Scientific* and *Tokyo Chemical Industry* (TCI) and used without further purification. All phosphoramidite syntheses were carried out using extra-dry AcroSeal solvents purchased from Thermo Fisher Scientific.

Flash chromatography

Flash chromatography was performed using silica gel (40-63 μm, Sigma Aldrich). Columns were packed under pressure and products were loaded dissolved in the eluent. Purification was carried out under pressure.

Thin layer chromatography

Thin layer chromatography (TLC) was carried out using aluminium plates coated with silica gel (silica gel 60, F254, layer thickness 0.250 mm). Compounds were visualized under UV light at λ = 254 nm or λ = 366 nm.

Nuclear magnetic resonance spectroscopy (NMR)

NMR-spectra were recorded on Bruker Avance 400 (400 MHz for ^{1}H and 101 MHz for ^{13}C) and Bruker Avance 500 (500 MHz for ^{1}H and 126 MHz for ^{13}C) at the Institute of Organic Chemistry, Karlsruher Institute of Technology (KIT). Chemical shifts (δ) are reported in parts per million (ppm) relative to the residual solvent signal. Coupling constants (J) are reported in hertz (Hz).

Mass spectrometry ESI

High resolution mass spectra were recorded using a Q exactive Plus Orbitrap mass spectrometer (Thermo Fisher) with an electrospray ionization (ESI) source. Measurements were performed by the analytical department of the Institute of Organic Chemistry at the Karlsruhe Institute of Technology.

MALDI-TOF MS

MALDI-TOF mass spectra were performed at a Shimadzu Axima Confidence mass spectrometer. The following matrice was used:

- 3-hydroxypicolinic acid (HPA) dissolved in MeCN/H_2O (1:1, v/v). Diammonium hydrogen citrate (0.44 M, aqueous) was added at a ratio 9:1 (v/v).

Optical spectroscopy

Oligonucleotide melting temperatures were determined using a Varian Cary 100 Bio spectrometer. Fluorescence quantum yields were determined at a Quantaurus QY C11347 from Hamamatsu and absorption spectra were recorded on a Perkin Elmer Lambda 750 absorption spectrometer equipped with a Peltier element PTP 6+6 Peltier system or on a Cary 3500 Multicell UV/Vis spectrophotometer (Agilent Technologies) using a Peltier temperature control. The spectra were corrected against the corresponding solvent spectra at measured at 20 °C.

Fluorescence Spectroscopy

Fluorescence measurements were conducted using a FluoroMax spectrofluorometer (HORIBA Jobin Yvon). Solvent background contributions were removed by blank substraction using the solvent spectrum.

2. Oligonucleotide synthesis

Oligonucleotides were synthesized under argon on an H-6 DNA/RNA synthesizer (K&A Laborgeräte) using controlled pore glass (CPG, 500 Å, 1 μmol) as solid phase. Phosphoramidites and CPG were obtained from Sigma Aldrich, ChemGenes, Proligo and GlenResearch. Synthesized phosphoramidites were dissolved in MeCN of a concentration of 100 μM. After DNA-synthesis, columns were dried in vacuum followed by deprotection. Deprotection were carried out with NH_4OH at 55 °C for 16 h. Solvents were removed under reduced pressure and the oligonucleotides were prepurified by by Glen-Pak™ DNA Purification Cartridges (GlenResearch). Purification was completed by HPLC-chromatography.

Oligonucleotide concentrations were determined from their absorbance at 260 nm using a NanoDrop ND-100 spectrophotometer.

$\epsilon_{260} = (A*\epsilon A + T*\epsilon T + C*\epsilon C + G*\epsilon G) * 0.9 + F*\epsilon F$

ϵ: molar extinction coefficient

X: number of nucleotides (A, T, C, G)

F: number of fluorophores TO or DB

The molar extinction coefficient of thiazole orange is 9400 $Lmol^{-1}cm^{-1}$ and of dibenzothiazole is 6900 $Lmol^{-1}cm^{-1}$.

Hybridization was performed at 90 C in 10 mM NaP_i buffer with 250 mM NaCl at pH 7 in ddH_2O. Concentrations of oligonucleotides was 2.5 μmol. Fluorophore-modified counterstrands were added at 1.0 and non-modified counter strands were added at 1.1 equivalents.

High Performance Liquid Chromatography (HPLC)

Purification of synthesized oligonucleotides was performed by HPLC on a Dionex Ultimate 3000 system (Thermo Fisher Scientific) equipped with an autosampler, column oven and diode-array detector. Data were collected using Chromeleon 7 software. Semi-preparative purification were carried out on a Discovery® BIO Wide Pore C18 column (250 x 10 mm, 5 μm, Supelco) with a flow rate of 2.5 mL/min. Elution was performed at 40 °C using a gradient of acetonitrile (0-20 %) in 50 mM Ammonium acetate (60 min). Analytical HPLC was performed on a VDSpher OptiBio PUR 300 S18-SE column (250 x 4.6 mm, 5 μm) with a flow rate of 1.0 mL/min. Elution was carried out at 40 °C using a gradient of acetonitrile (0-30 %) in 50 mm ammonium acetate over 70 min.

3. Synthesis of TO and DB building blocks 7 und 11

Compound **1**

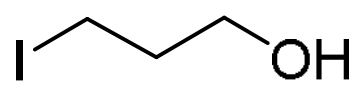

C_3H_7IO
MW = 185.99 g/mol

9.45 g 3-Chloropropanol (0.10 mol, 1.00 eq.) and 75 g NaI (0.50 mol, 5.00 eq.) were dissolved in 100 mL dry acetone and stirred for 16 h at 60°C. The solid was filtered off and the solvent was removed under reduced pressure. The residue was mixed 100 mL of a 1:1 mixture of diethyl ether and hexane and filtered again. The solution was washed with 50 mL each of a dilute sodium thiosulfate solution, with water and then with a saturated aqueous sodium chloride solution. The organic phase was dried over $MgSO_4$, the solid was filtered off and the solvent was removed under reduced pressure. Product **1** (11.2 g, 60.0 mmol, 60%) were obtained as slight yellow liquid.

^{1}H-NMR: (400 MHz, $CDCl_3$): δ (ppm) = 3.72 (t, *J* = 5.9 Hz, 2H, CH_2OH), 3.29 (t, *J* = 6.7 Hz, 2H, CH_2I), 2.03 (m, 2H, CH_2).

The analytical data are consistent with the literature.[29]

Compound **2**

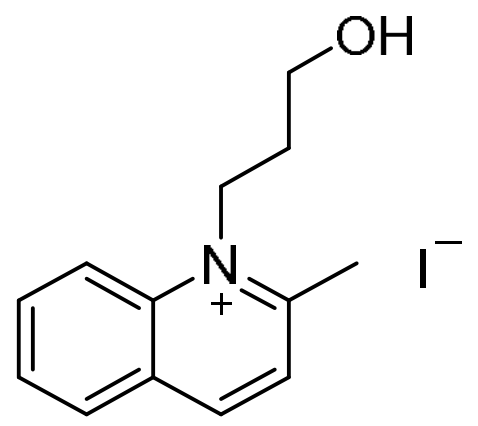

$C_{13}H_{16}INO$
MW = 329.19 g/mol

Under argon, 5.40 mL 2-methylquinoline (5.73 g, 39.8 mmol, 1.00 eq.) and 5.51 mL **1** (11.2 g, 60.0 mmol, 1.50 eq.) were dissolved in 25 mL 1,4-dioxane and stirred for 72 h at 101 °C. After cooling, 40 mL of diethyl ether was added and the precipitate was filtered off. The solid was washed three times with 30 mL diethyl ether and then dried. Product **2** was obtained as light gray solid (5.32 g of 16.7 mmol, 42 %).

^{1}H-NMR: (400 MHz, DMSO-d$_6$): δ (ppm) = 9.10 (d, *J* = 8,5 Hz, 1H, arom.), 8.60 (d, *J* = 9.0 Hz, 1H, arom.), 8.41 (d, *J* = 8.0 Hz, 1H, arom.), 8.23 (t, *J* = 7.8 Hz, 1H, arom.), 8.12 (d, *J* = 8.6 Hz, 1H, arom.), 7.99 (t, *J* = 7.6 Hz, 1H, arom.), 5.00 (m, 2H, CH_2N), 3.64 (t, *J* = 5.6 Hz, 2H, CH_2OH), 3.13 (s, 3H, CH_3), 2.08 (m, 2H, CH_2).

The analytical data are consistent with the literature.[30]

Compound **3**

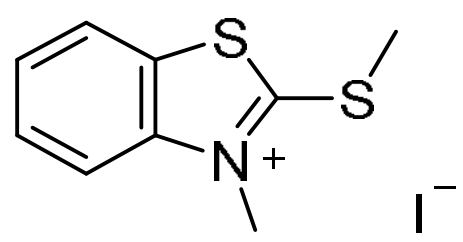

$C_9H_{10}INS_2$
MW = 323.21 g/mol

6.00 g methylthiobenzothiazole (33.1 mmol, 1.00 eq.) and 8.20 mL methyl iodide (18.7 g, 132 mmol, 4.00 eq.) were dissolved in 20 mL DMF and stirred for 24 h at 60 °C. The reaction mixture was then diluted with 20 mL ethyl acetate. The precipitate was filtered off and dried under reduced pressure. Product **3** (7.66 g, 23.7 mmol, 72 %) was obtained a white solid.

^{1}H-NMR: (400 MHz, DMSO-d$_6$): δ (ppm) = 8.41 (d, J = 8.2 Hz, 1H, arom.), 8.20 (d, J = 8.4 Hz, 1H, arom.), 7.85 (ddd, J = 8.5, 7.3, 1.0 Hz, 1H, arom.), 7.73 (ddd, J = 8.2, 7.3, 1.0 Hz, 1H, arom.), 4.12 (s, 3H, CH_3N), 3.13 (s, 3H, CH_3S).

The analytical data are consistent with the literature.[31]

Compound **4**

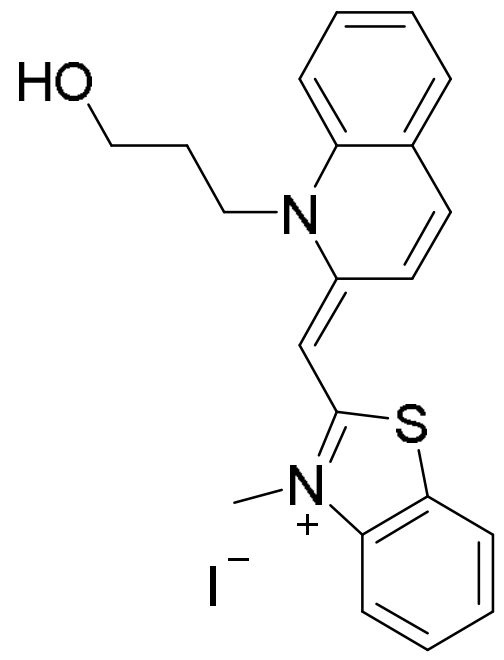

$C_{21}H_{21}IN_2OS$
MW = 476.48 g/mol

2.03 g **2** (6.17 mmol, 1.00 eq.) and 2.29 g **3** (7.10 mmol, 1.10 eq.) were dissolved in 50 mL of a 1:1 solvent mixture of MeOH and DCM. Then 2.68 mL of triethylamine (1.96 g, 18.1 mmol, 3.00 eq.) was added. Using wash bottles with an aqueous NaOH solution, the reaction was stirred for 24 h at r.t.. The reaction was then diluted with 50 mL diethyl ether and the precipitate was filtered off. The solid was washed twice with 30 mL diethyl ether and then washed with water and dried in vacuo. Product **4** was obtained as red solid (746 mg, 1.56 mmol, 25 %) was obtained.

^{1}H-NMR: (400 MHz, DMSO-d$_6$): δ (ppm) = 8.46 (d, *J* = 9.4 Hz, 1H, arom.), 8.16 (d, *J* = 8.9 Hz, 2H, arom.), 8.04 (m, 3H, arom.), 7.92 (t, *J* = 8.0 Hz, 1H, arom.), 7.81 (d, *J* = 8.3 Hz, 1H, arom.), 7.62 (m, 2H, arom.), 7.43 (t, *J* = 7.6 Hz, arom.), 1H), 6.33 (s, 1H, CH), 5.18 (t, *J* = 4.7 Hz, 1H, OH), 4.70 (s, 1H, CH_2N), 3.92 (s, 3H; CH_3), 3.71 (q, *J* = 5.2 Hz, 2H, CH_2O); 2.00 (m, 2H, CH_2).

^{13}C-NMR (101 MHz, DMSO-d$_6$) δ (ppm) = 162.32, 152.82, 141.19, 140.48, 139.49, 134.08, 130.13, 128.56, 126.18, 125.30, 124.62, 123.89, 123.34, 119.06, 117.45, 113.63, 86.15, 58.06, 58.02, 46.47, 34.39.

HR-ESI-MS: m/z (calculated) = 349.1361 [M$^+$]; m/z (found) = 349.1369 [M$^+$].

Compound **6**

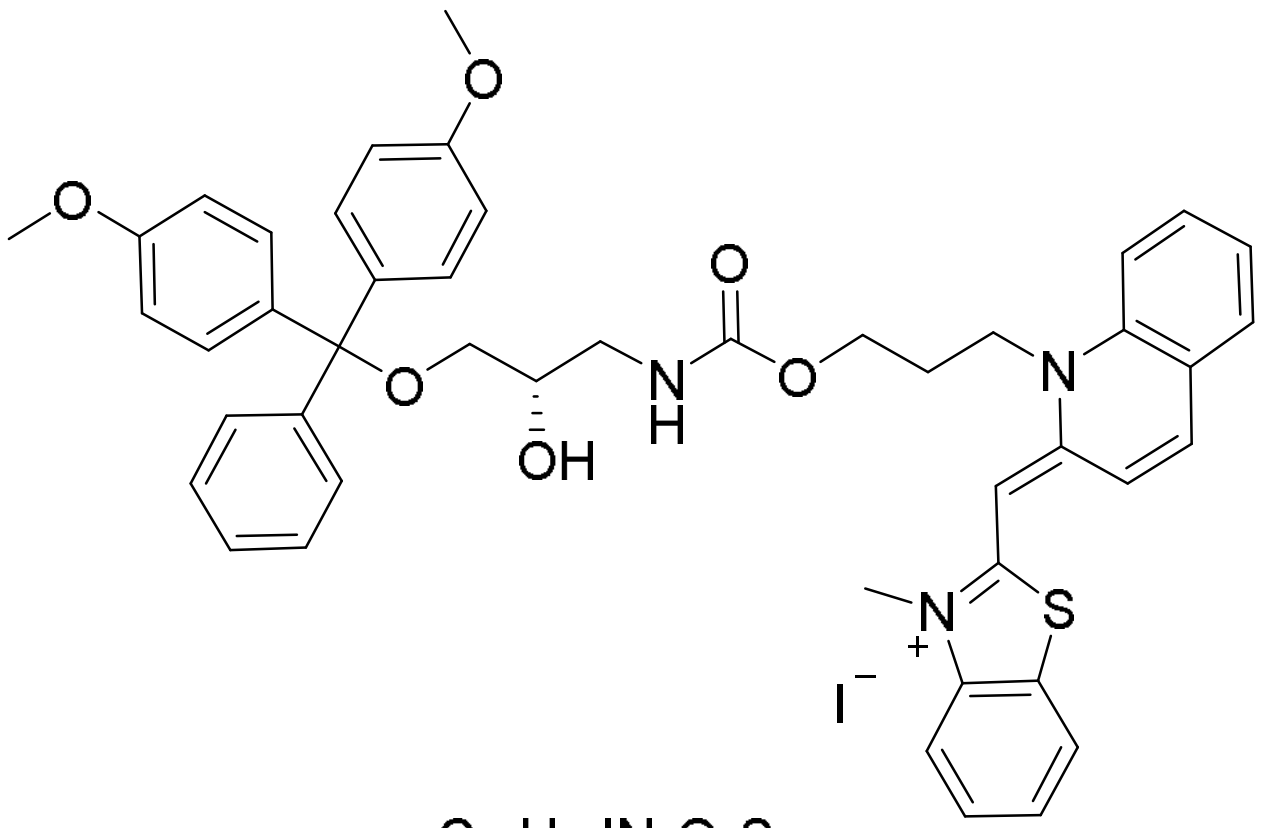

$C_{46}H_{46}IN_3O_6S$
MW = 895.86 g/mol

Under argon atmosphere, 746 mg **4** (1.56 mmol, 1.00 eq.) and 303 mg 1,1'-carbonyldiimidazole (1.87 mmol, 1.2 eq.) were dissolved in 25 mL DMF and stirred for 3 h at r.t.. Subsequently, 736 mg **5**[28] (1.87 mmol, 1.2 eq.) were added and stirred for 72 h at r.t.. The solvent was then removed under reduced pressure and the crude product was column chromatographed with methanol in DCM (0 -5 %). Product **6** was 726 mg (0.81 mmol, 52 %) of a red solid.

TLC (DCM/MeOH 9:1) R*f* = 0.4.
^{1}H-NMR: (500 MHz, DMSO-d$_6$): δ (ppm) = 8.45 (m, 1H, arom. TO), 8.11-8.00(m, 4H, arom. TO), 7.91 (m, 1H, arom.), 7.76 (m, 1H, arom. TO), 7.61 (m, 2H, arom. TO), 7.47-7.41 (m, 1H, arom. TO), 7.41-7.36 (m, 2H, arom. DMT), 7.28 (m, 1H, arom. TO), 7.27-7.09 (m, 7H, arom. DMT), 6.89-6.79 (m, 4H, arom. DMT), 4.99 (d, J = 5.3 Hz, 1H, OH), 4.62 (t, 2H, CH_2N), 4.24 (t, J = 6.1 Hz, 2H, CH_2O), 3.91 (s, 3H, CH_3N), 3.74 (m, 1H,CHOH), 3.69 (d, J = 2.5 Hz, 6H, OCH_3), 3.24 (m, 1H, $NHCH_2$), 3.05-2.91 (m, 2H, CH_2ODMT), 2.88 (m, 1H, $NHCH_2$), 2.17 (s, 2H, CH_2).
^{13}C-NMR: (126 MHz, DMSO-*d*$_6$) δ (ppm) = 162.16, 157.97, 156.23, 152.33, 145.05, 140.70, 140.08, 138.87, 135.76, 135.73, 133.69, 129.73, 128.14, 127.72, 126.54, 125.71, 124.95, 124.22, 123.50, 122.92, 118.63, 116.75, 113.15, 113.05, 85.43, 85.18, 72.27, 68.79, 65.69, 61.02, 60.25, 54.99, 54.92, 45.63, 45.36, 44.25, 33.90, 26.14.
HR-ESI-MS: m/z (calculated) = 768.3100 [M^+]; m/z (found) = 768.3102 [M^+].

Compound **7**

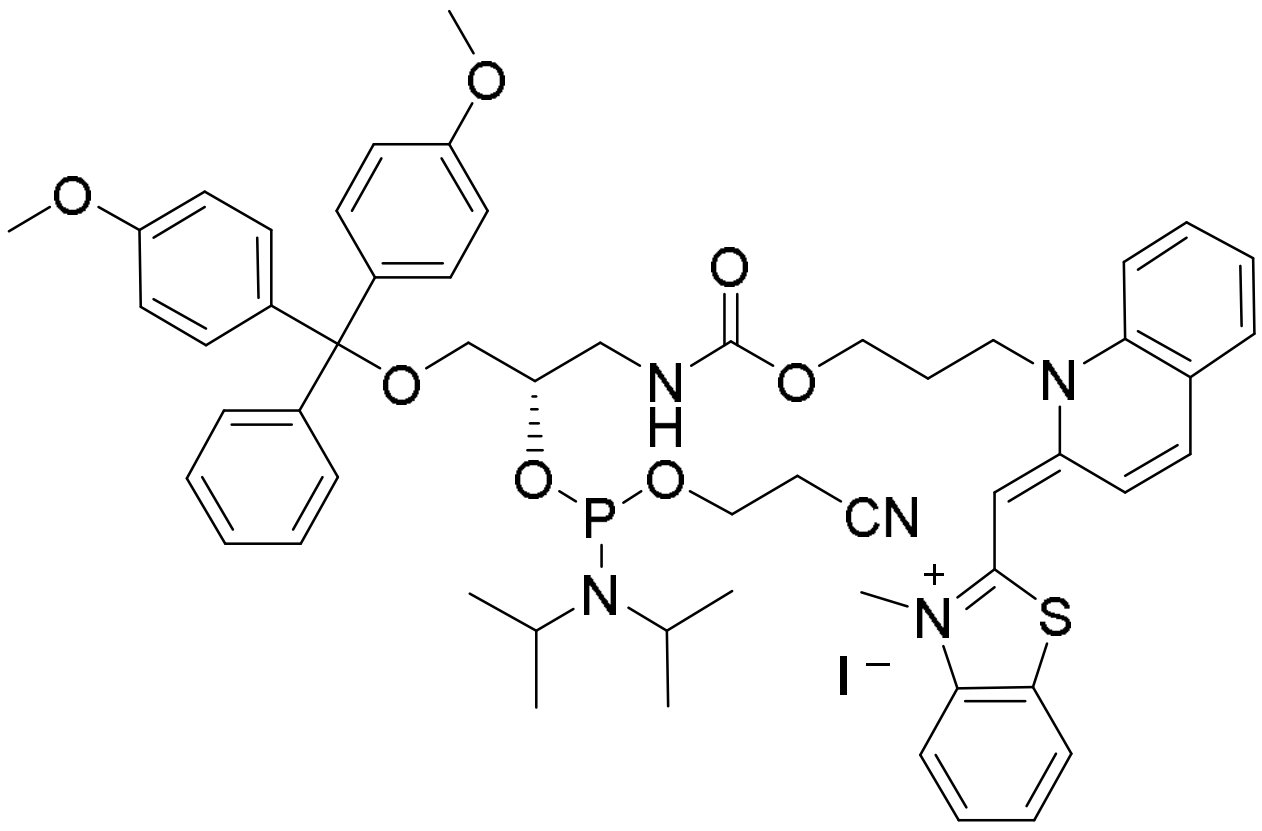

$C_{55}H_{63}N_5O_7IPS$
MW = 1096.08 g/mol

150 mg **6** (0.17 mmol, 1.00 eq.) was dissolved under argon in 10 mL DCM and mixed with 90 µL (0.51 mmol, 3.00 eq.) dry diisopropylamine and 52.5 µL (0.24 mmol, 1.40 eq.) 2-cyanoethyl-N,N-diisopropylchlorophosphoramidite. The reaction solution was stirred for 3 h at r.t. before it was diluted with a freshly prepared saturated aqueous $NaHCO_3$ solution. It was then dried over Na_2SO_4, filtered and the solvent was removed under reduced pressure. A dark red solid was obtained, which was dissolved in 1.7 mL MeCN and was immediately prepared for automated DNA synthesis.

TLC (DCM/MeOH 9:1) R*f* = 0.5.
^{31}P-NMR: (151 MHz, DMSO-d6): δ (ppm) = 147.91, 148.47.
HR-ESI-MS: m/z (calculated) = 968.4170 [M^+]; m/z (found) = 968.4180 [M^+].

Compound **8**

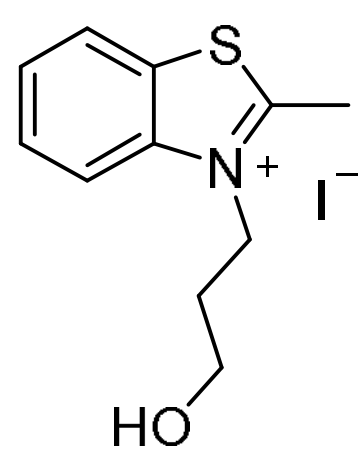

$C_{11}H_{14}INOS$
MW = 335.20 g/mol

15.0 mL 2- Methylbenzothiazole (17.6 g, 118 mmol, 2.00 eq.) and 11.0 g **1** (59.0 mmol, 1.00 eq.) were dissolved in 35 mL acetonitrile. The reaction mixture was heated under reflux for 72 h, then allowed to cool to room temperature and stored at 4 °C overnight. The precipated solid was collected by filtration and washed with cold diethyl ether (3 x 20 mL). Drying under reduced pressure afforded 8.72 g of product **8** as a white solid (26.0 mmol, 44%).

^{1}H-NMR: (400 MHz, DMSO-d$_6$): δ (ppm) = 8.45 (dd, J = 8.2, 1.2 Hz, 1H, arom.), 8.31 (d, J = 8.5 Hz, 1H, arom.), 7.90 (m, 1H, arom.), 7.85 – 7.76 (m, 1H, arom.), 4.83 (t, J = 4.9 Hz, 1H, OH), 4.78 (t, J = 7.2 Hz, 2H, NCH$_2$), 3.52 (q, J = 5.3 Hz, 2H, OCH$_2$), 3.22 (s, 3H, CH$_3$), 2.10 0 1.98 (m, 2H, CH$_2$).

The analytical data are consistent with the literature.[4]

Compound **9**

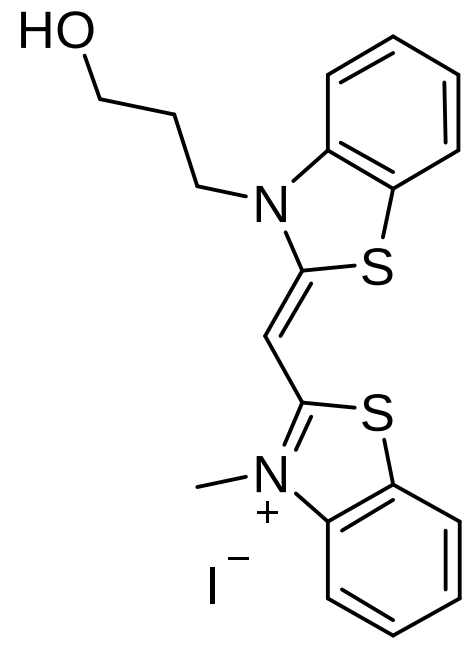

$C_{19}H_{19}IN_2OS_2$
MW = 482.40 g/mol

500 mg **8** (1.49 mmol, 1.00 eq.) and 530 mg **3** (1.64 mmol, 1.10 eq.) were dissolved in 50 mL ethanol. 0.62 mL triethylamine (452 mg, 4.47 mmol, 3.00 eq.) was added, and the reaction mixture was stirred at 80 °C for 1 h and subsequently at room temperature for 16 h. The resulting precipitate was collected by filtration. The solid was washed twice with ethanol (2 x 30 mL), followed by diethyl ether and dried under reduced pressure to afford 535 mg of product 9 as yellow solid (1.11 mmol, 75 %).

^{1}H-NMR: (400 MHz, DMSO-d$_6$): δ (ppm) = 8.18 (d, J = 9.6 Hz, 2H, arom.), 7.83 (dt, J = 9.6 Hz, 2H, arom.) 7.66 – 7.57 (m, 2H, arom.), 7.48 – 7.39 (m, 2H, arom.), 6.77 (s, 1H, CH), 4.97 (t, J = 4.8 hz, 1H, OH), 4.62 (t, J = 6.9 Hz, 2H, NCH$_2$), 3.98 (s, 3H, CH$_3$), 3.55 (q, J = 5.4 Hz, 2H, OCH$_2$), 1.99 (p, J = 66 Hz, 2H, CH$_2$).

^{13}C-NMR: (101 MHz, DMSO-*d*$_6$) δ (ppm) = 161.93, 161.55, 140. 59, 140.13, 128.49, 128.49, 128.42, 124.80, 124.70, 123.48, 123.40, 113.62, 82.71, 57.12, 43.50, 34.08, 29.93.

HR-ESI-MS: m/z (calculated) = 355.0933 [M$^+$]; m/z (found) = 355.0930 [M$^+$].

Compound **10**

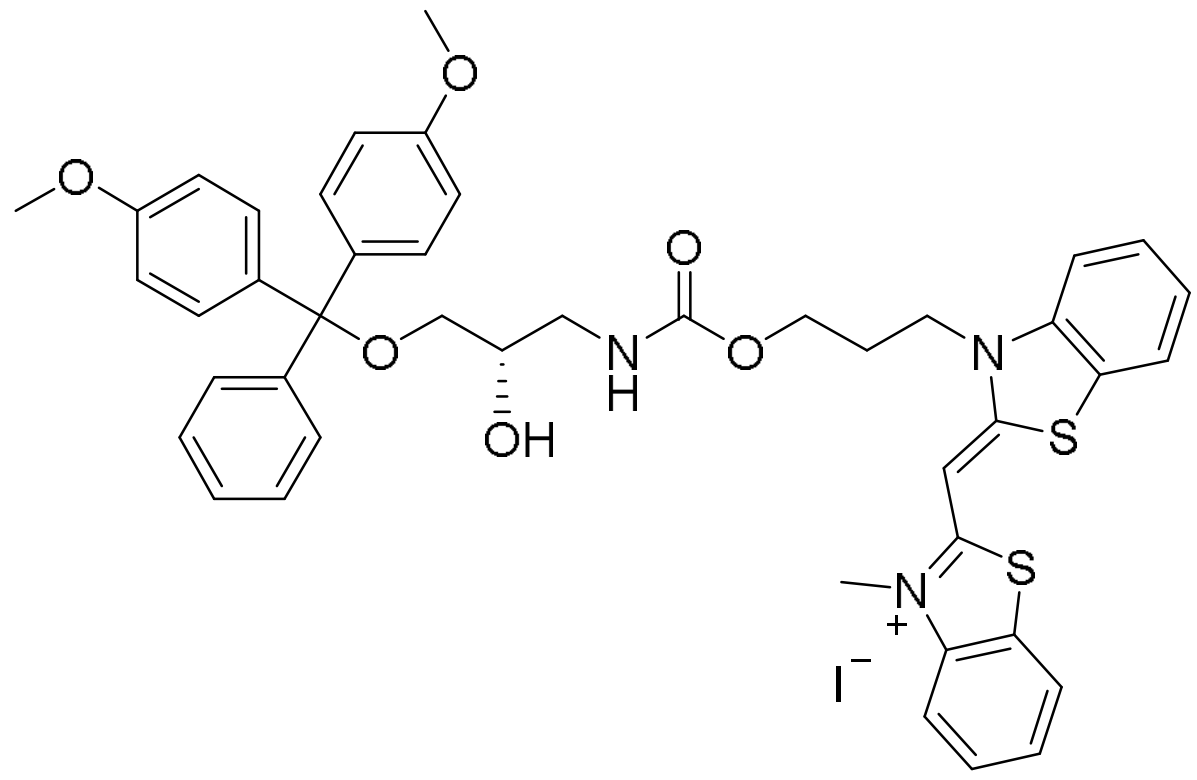

$C_{44}H_{44}IN_3O_6S_2$
MW = 901.88 g/mol

600 mg (1.24 mmol, 1.00eq.) of compound **9** was dissolved in 12 mL dry DMF under argon. 242 mg 1,1´-Carbonyldiimidazole (CDI) (1.49 mmol, 1.20 eq.) was added, and the reaction mixture was stirred at room temperature for 3 h. Subsequently, 586 mg of Linker **5**[28] (1.40 mmol, 1.20 eq.) was added, and stirring was continued at room temperature for an additional 48 h. The solvent was removed under reduced pressure at high vacuum, and the resulting crude product was purified by flash column chromatography using a gradient of MeOH in DCM (0-5%). 385 mg of product **10** was obtained as an orange solid (397 µmol, 32%).

DC (DCM/MeOH 9:1) R*f* = 0.45.

^{1}H-NMR: (400 MHz, DMSO-d$_6$): δ (ppm) = 8.23-8.17 (m, 2H, arom.-DB), 7.86-7.80 (m, 2H, arom.-DB), 7.70-7.60 (m, 2H, arom.), 7.51-7.43 (m, 2H, arom.-DB), 7.40-7.35 (m, 2H, arom.-DMT), 7.30-7.19 (m, 7H, arom.-DMT), 7.07-7.01 (m, 1H, arom.-DMT), 6.87-6.83 (m, 3H, arom.-DMT), 6.67 (s, 1H, CH-DB), 4,97 (d, J = 5.3 Hz, 1H, OH), 4,66 (t, t, J = 7.3 Hz, 2H, N-CH$_2$), 4.09 (t, J = 6.2 Hz, 2H, O-CH$_2$), 4.00 (s, 3H, CH$_3$), 3.73 (m, 1H, NH), 3.70 (s, 6H, DMT-CH$_3$), 3.24-3.14 (m, 1H, CH), 2.99-2.90 (m, 2H, NH-CH$_2$), 2.89-2.80 (m, DMT-CH$_2$), 2.17-2.07 (m, 2H, CH$_2$).

^{13}C-NMR: (101 MHz, DMSO-*d*$_6$) δ (ppm) = 162.25, 161.62, 157.96, 156.11, 145.03, 140.65, 140.06, 135.75, 135.72, 129.70, 128.57, 128.49, 127.71, 126.53, 124.93, 124.85, 124.79, 123.56, 123.44, 113.82, 113.54, 113.05, 85.17, 82.54, 68.73, 65.64, 60.73, 44.23, 43.28, 35.77, 34.20, 26.47.

HR-ESI-MS: m/z (calculated) = 774.2666 [M$^+$]; m/z (found) = 774.2669 [M$^+$].

Compound **11**

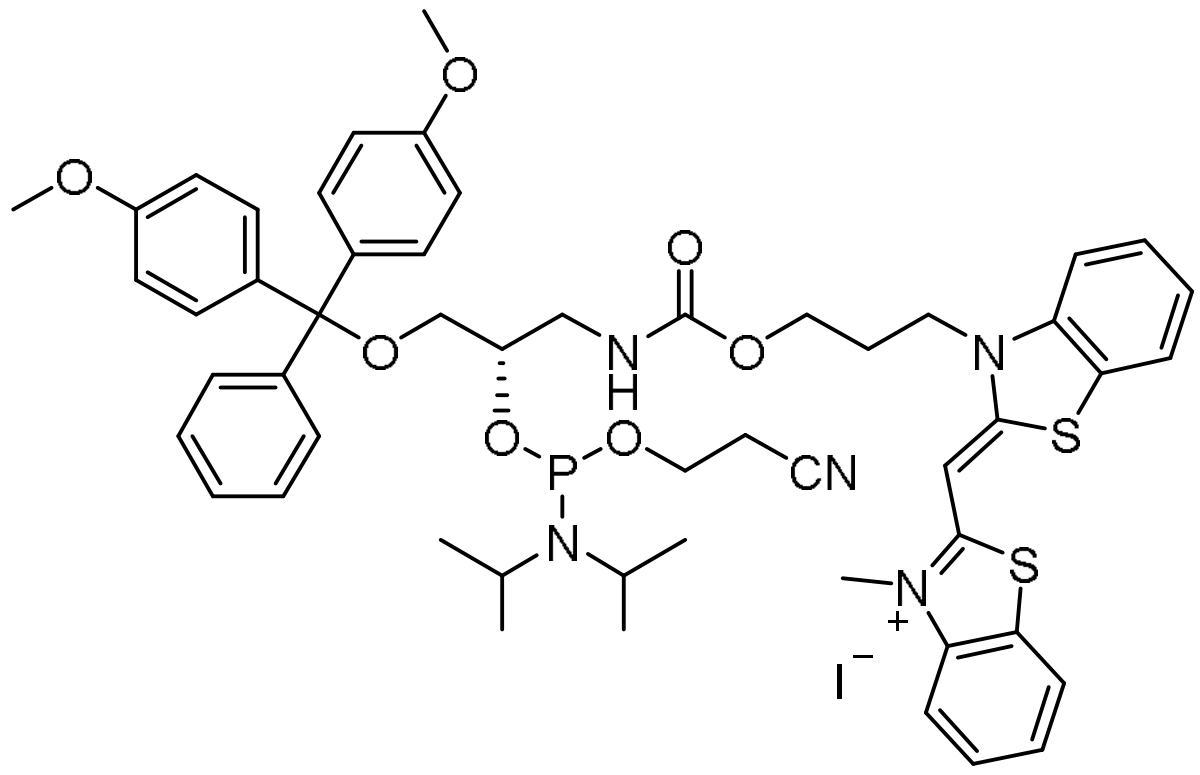

$C_{53}H_{61}IN_5O_7PS_2$
MW = 1102.10 g/mol

Under argon, 276 mg **10** (0.31 mmol, 1.00 eq.) was dissolved in 18 mL dry DCM. N,N-Diisopropylethylamine (160 µL, 0.92 mml, 3.00 eq.) was added, followed by dropwise addition of 102 µL 2-cyanoethyl-N,N-diisopropylchlorophosphoramidite (0.46 mml, 1.50 eq.). The reaction mixture was stirred at room temperature for 3 h. The reaction mixture was washed with saturated aqueous $NaHCO_3$ solution, followed by H_2O. The organic layer was dried over anhydrous Na_2SO_4, filtered, and the solvent was removed under reduced pressure to afford the product as an orange solid. The crude phosphoramidite was dissolved in 3.0 mL dry acetonitrile and used directly for automated DNA synthesis without further purification.

^{31}P-NMR: (151 MHz, DMSO-d$_6$): δ (ppm) = 147.86, 148.45.

HR-ESI-MS: m/z (calculated) = 974.3745 [M^+]; m/z (found) = 974.3742 [M^+].

4. Images of NMR spectra

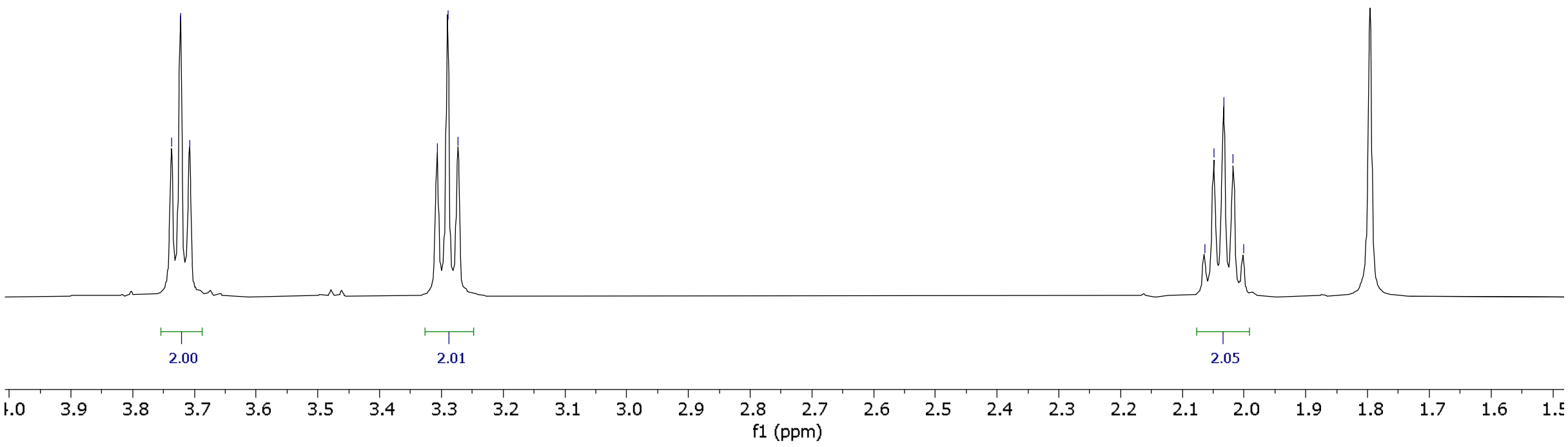

Figure S1: ^{1}H NMR spectrum of **1**.

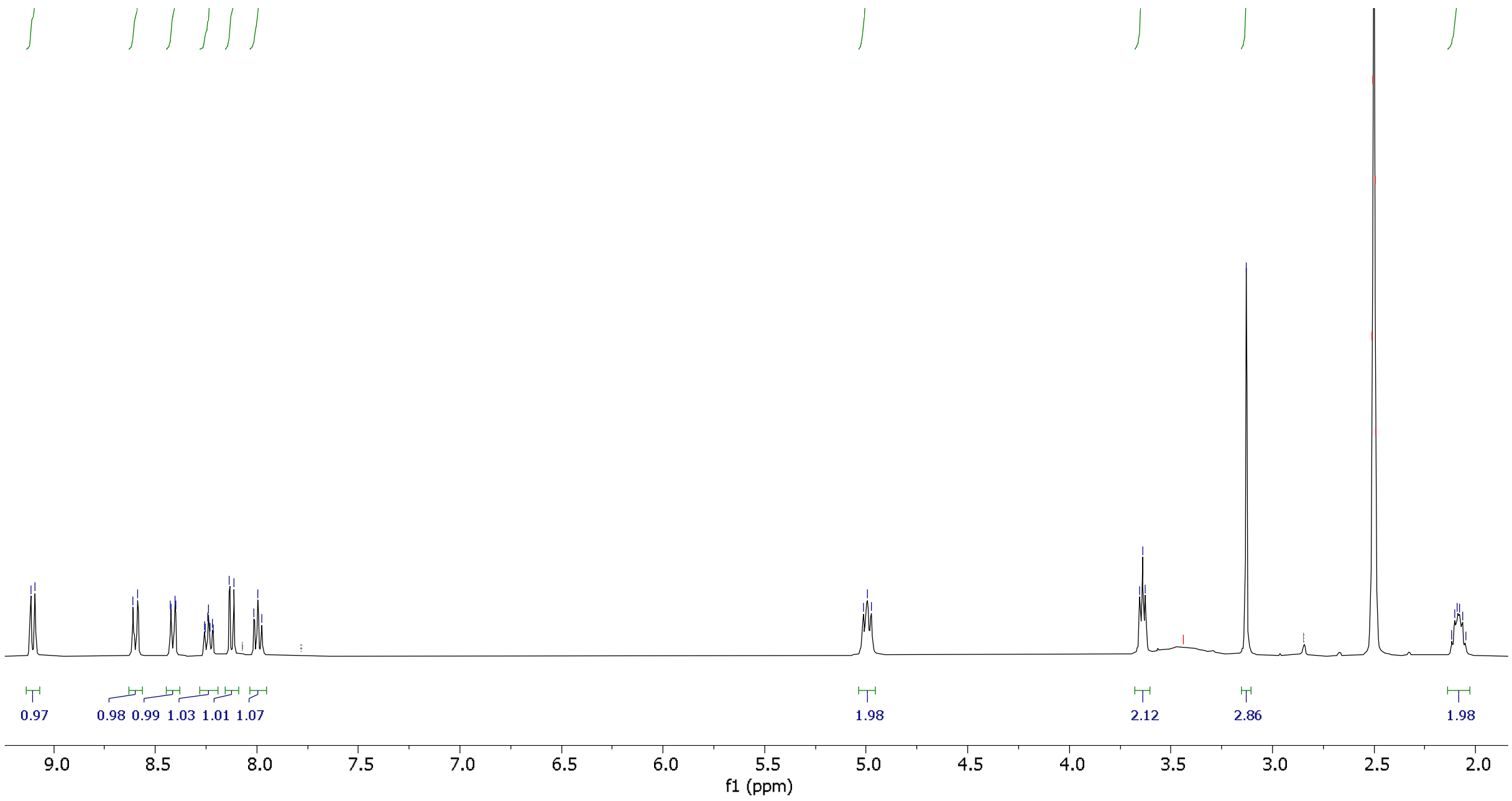

Figure S2: ^{1}H NMR spectrum of **2.**

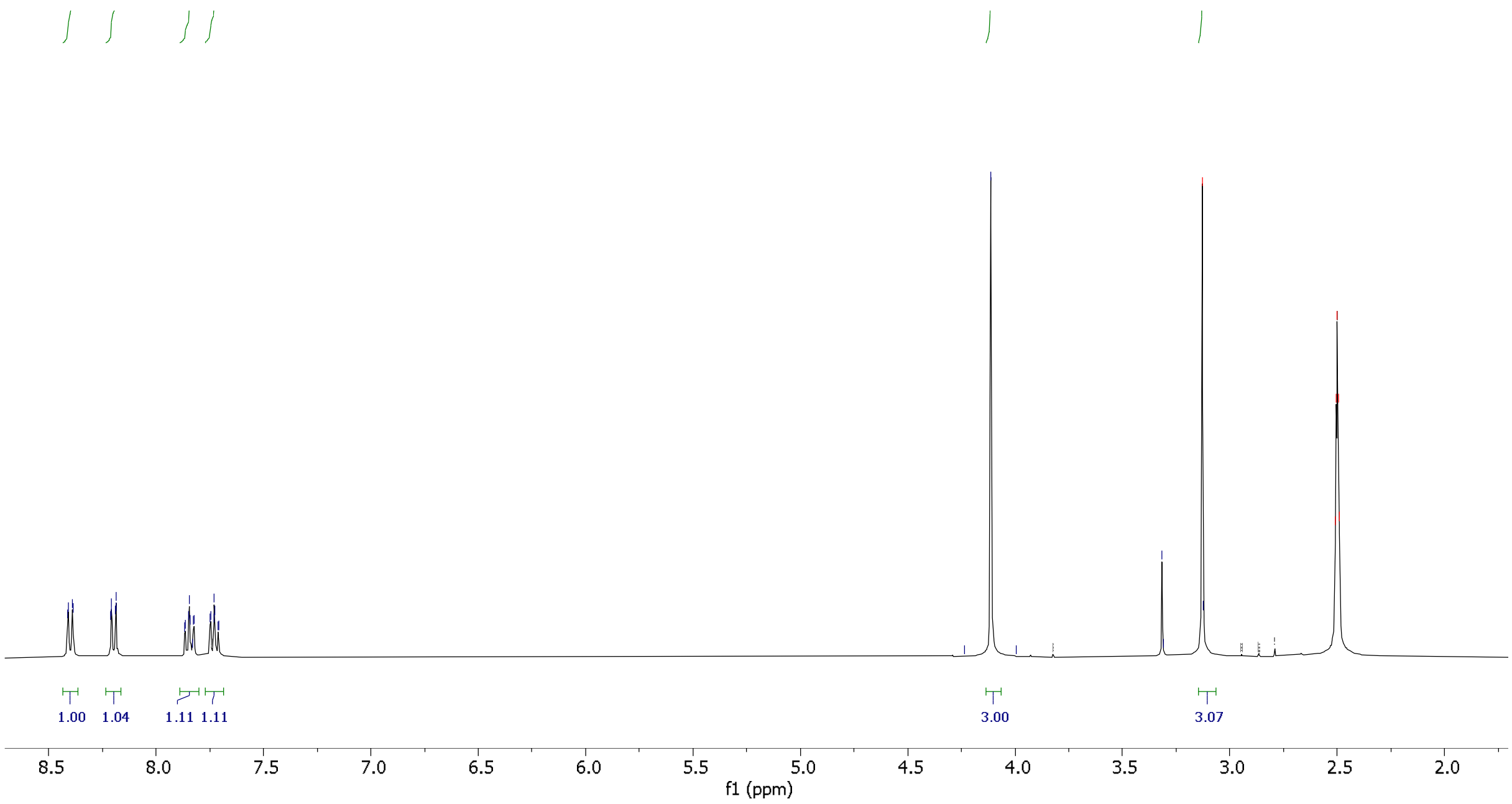

Figure S3: ^{1}H NMR spectrum of **3**.

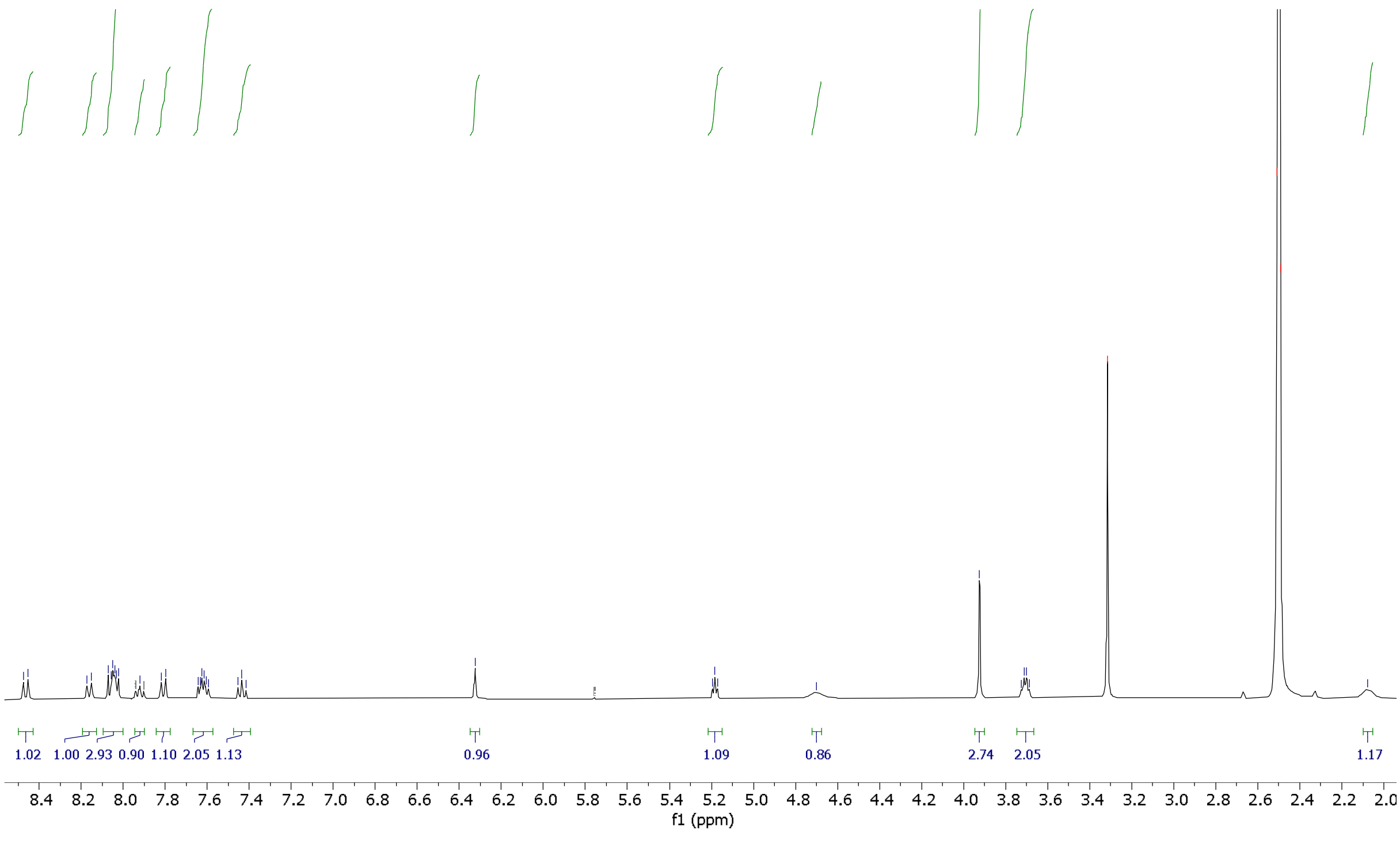

Figure S4: ^{1}H NMR spectrum of **4**.

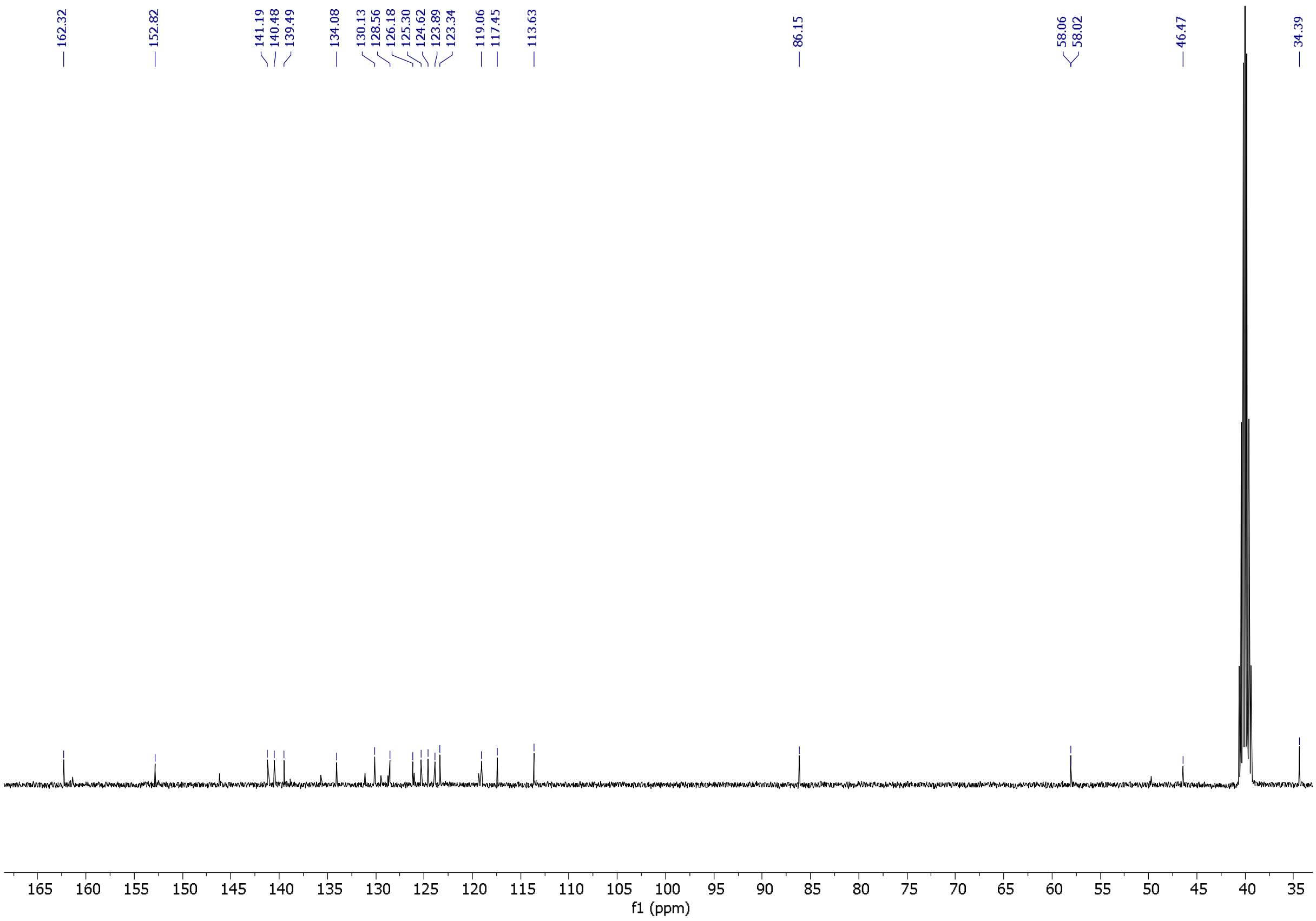

Figure S5: ^{13}C NMR spectrum of **4**.

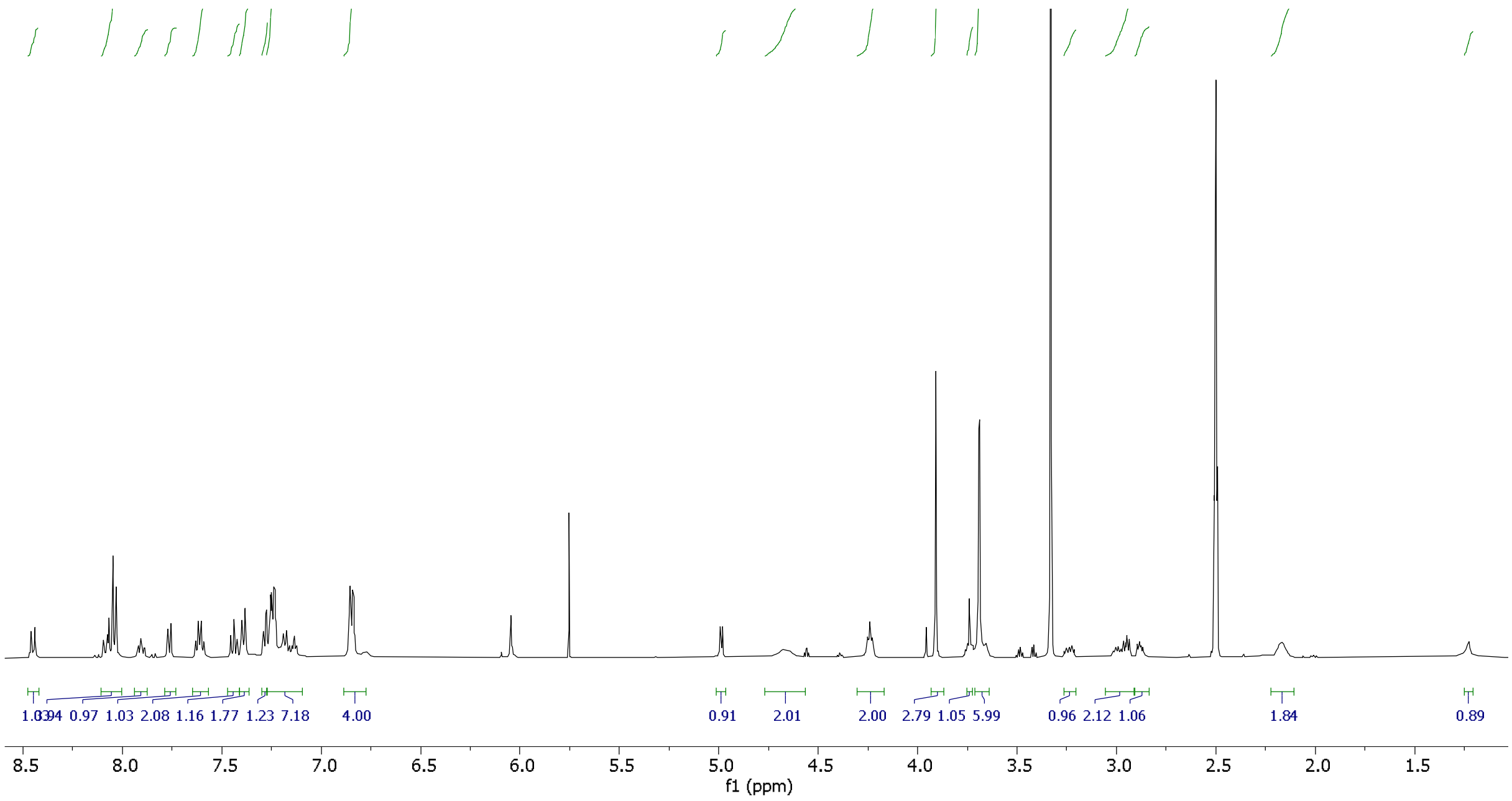

Figure S6: ^{1}H NMR spectrum of **6**.

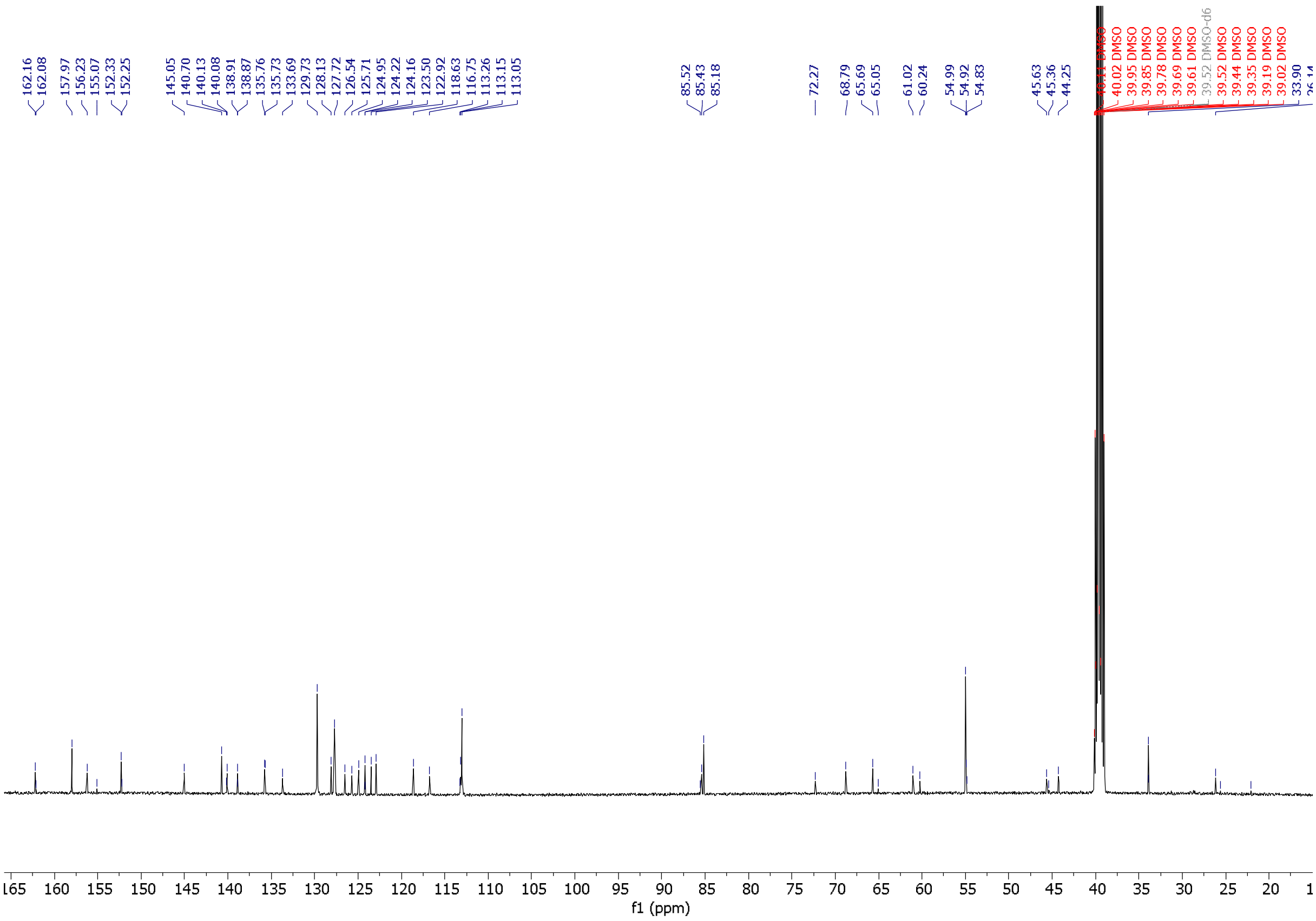

Figure S7: ^{13}C NMR spectrum of **6**.

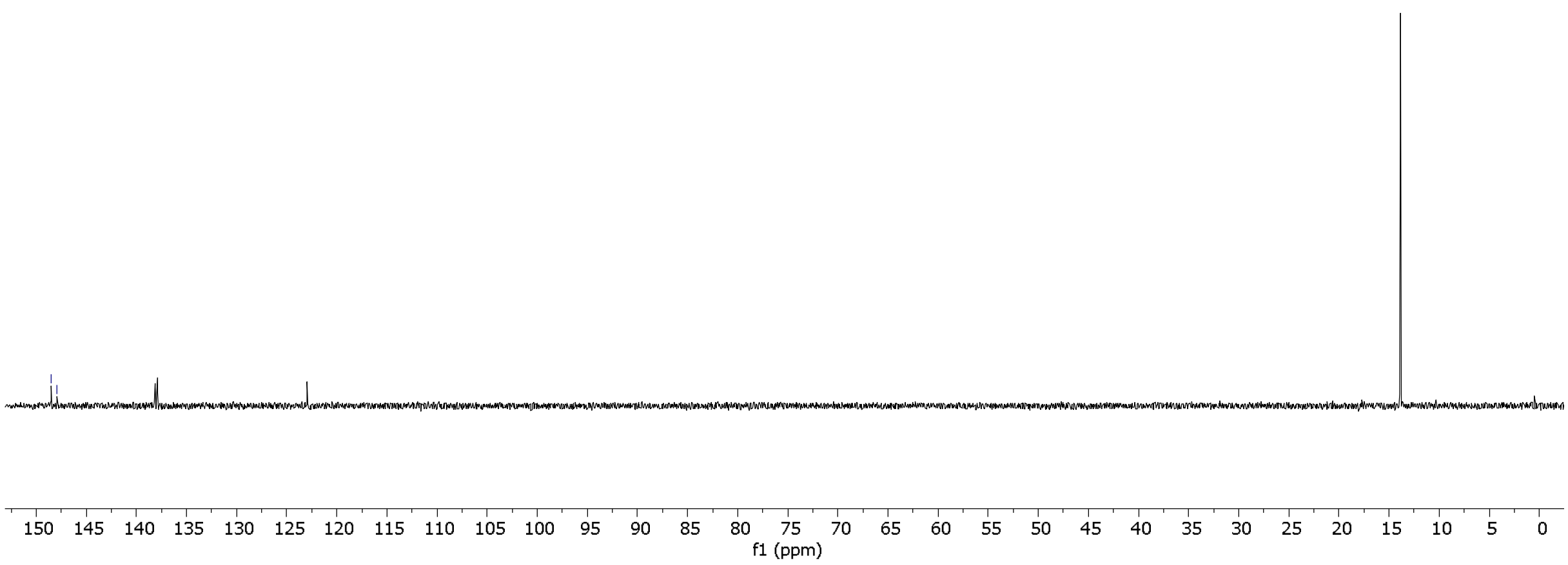

Figure S8: ^{31}P NMR spectrum of **7**.

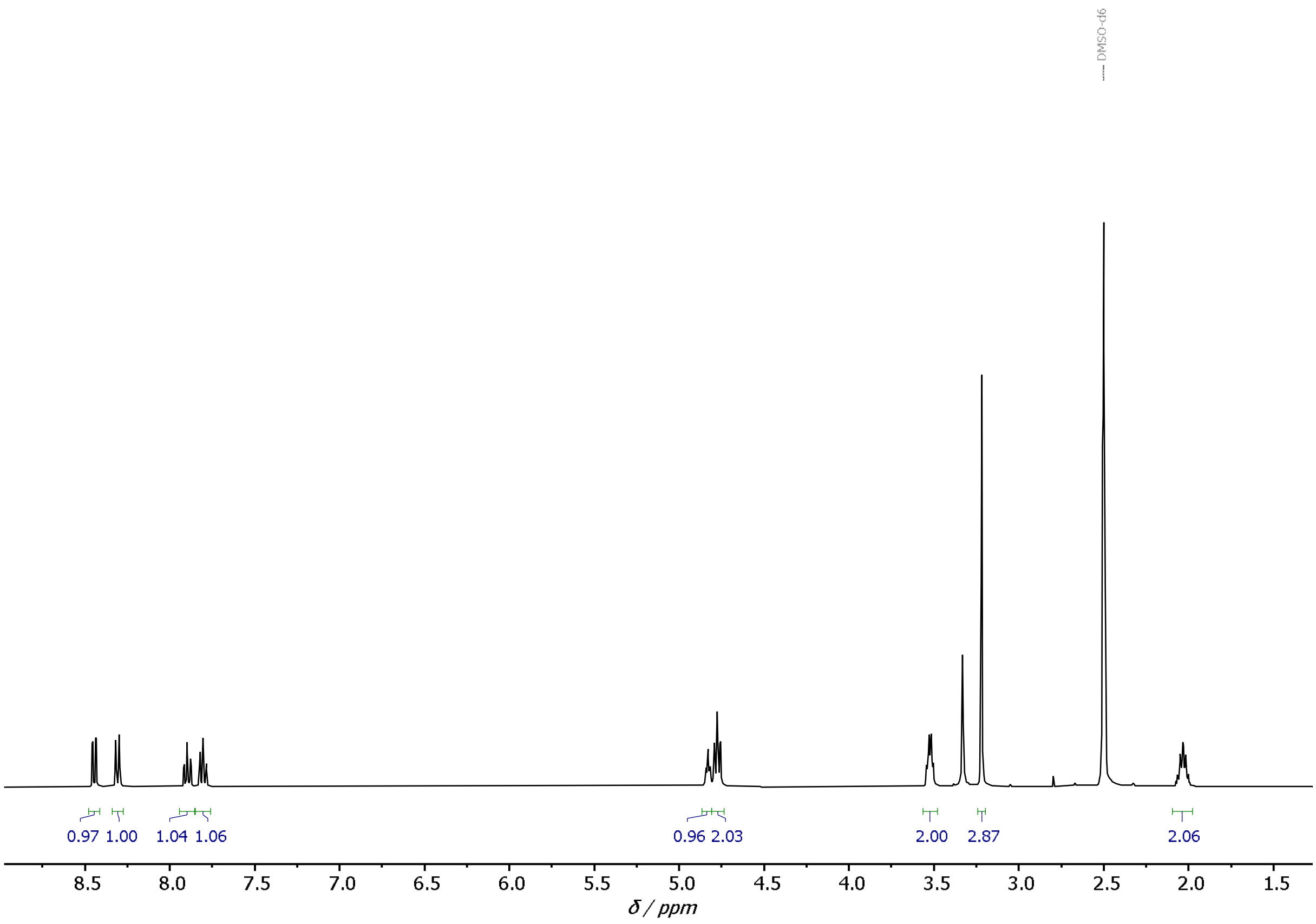

Figure S9: ^{1}H NMR spectrum of **8**.

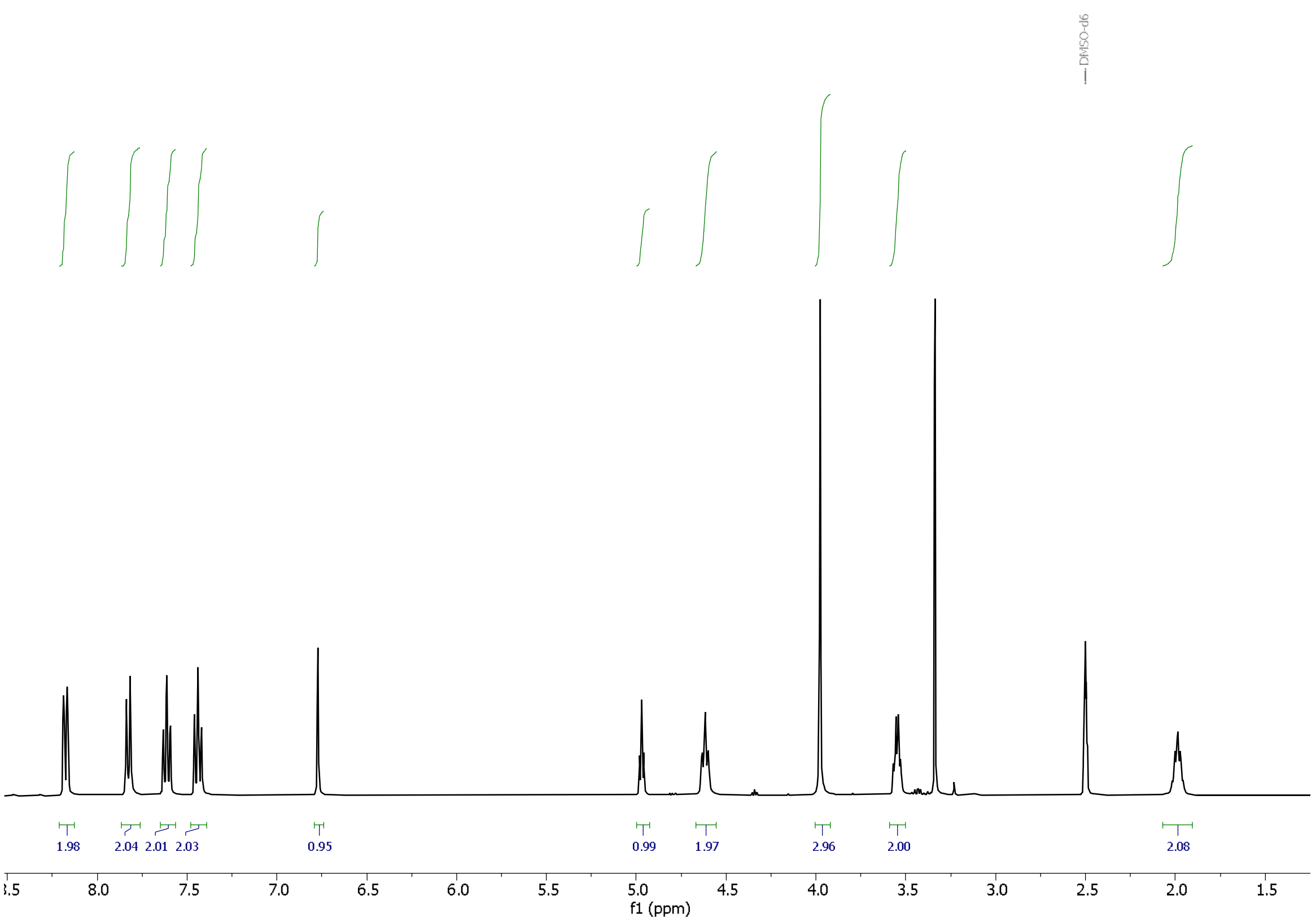

Figure S10: ^{1}H NMR spectrum of **9**.

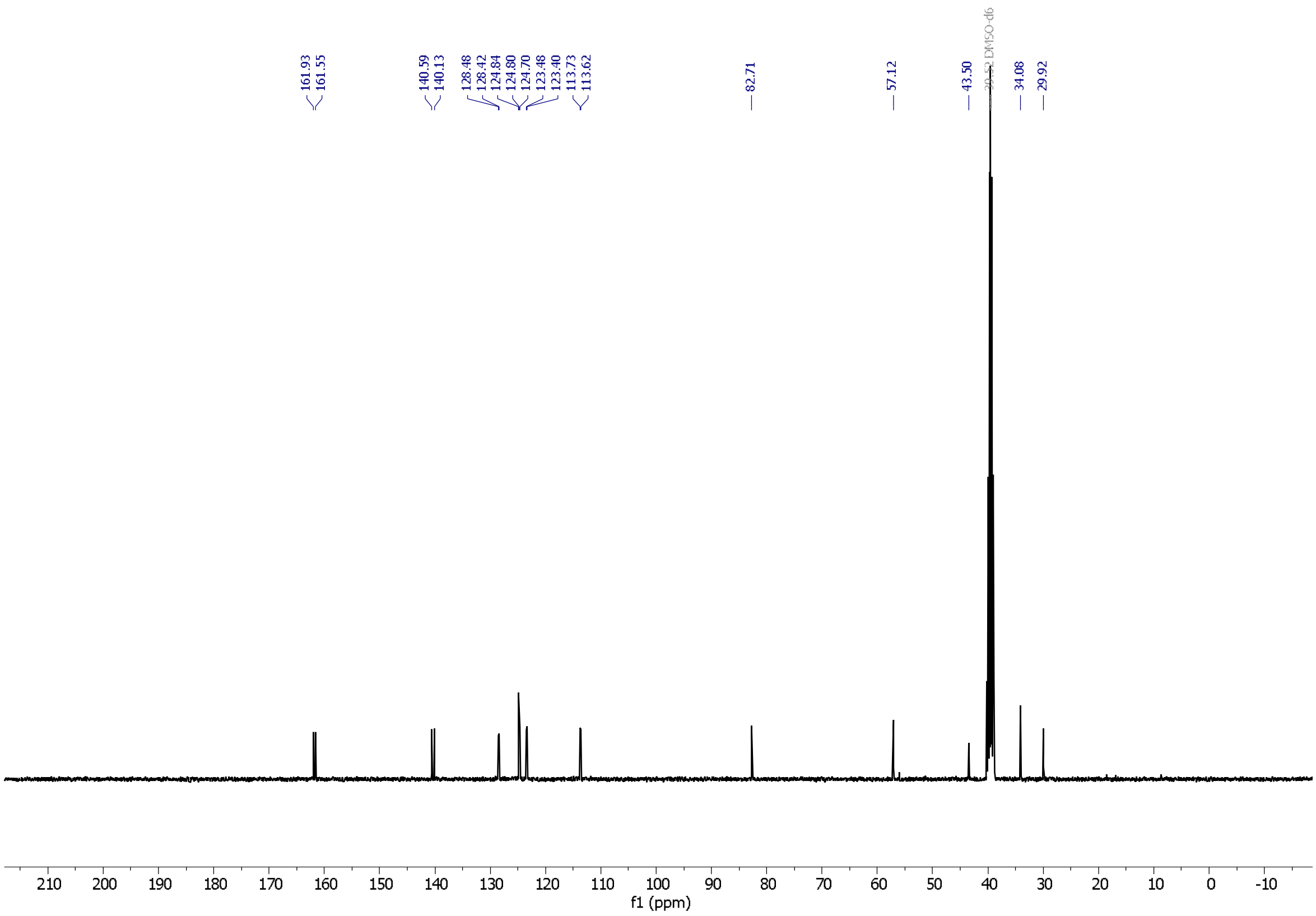

Figure S11: ^{13}C NMR spectrum of **9**.

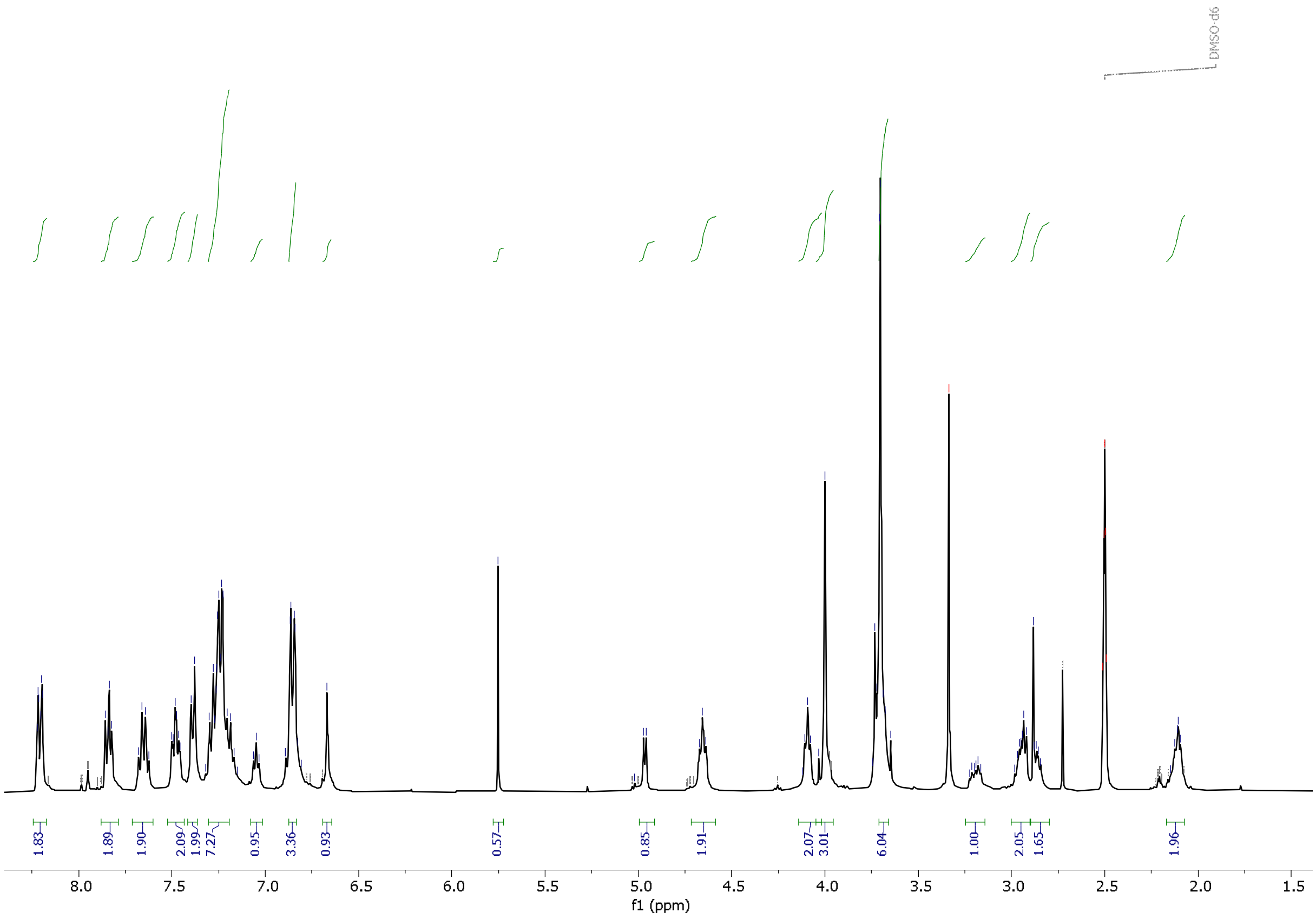

Figure S12: ^{1}H NMR spectrum of **10**.

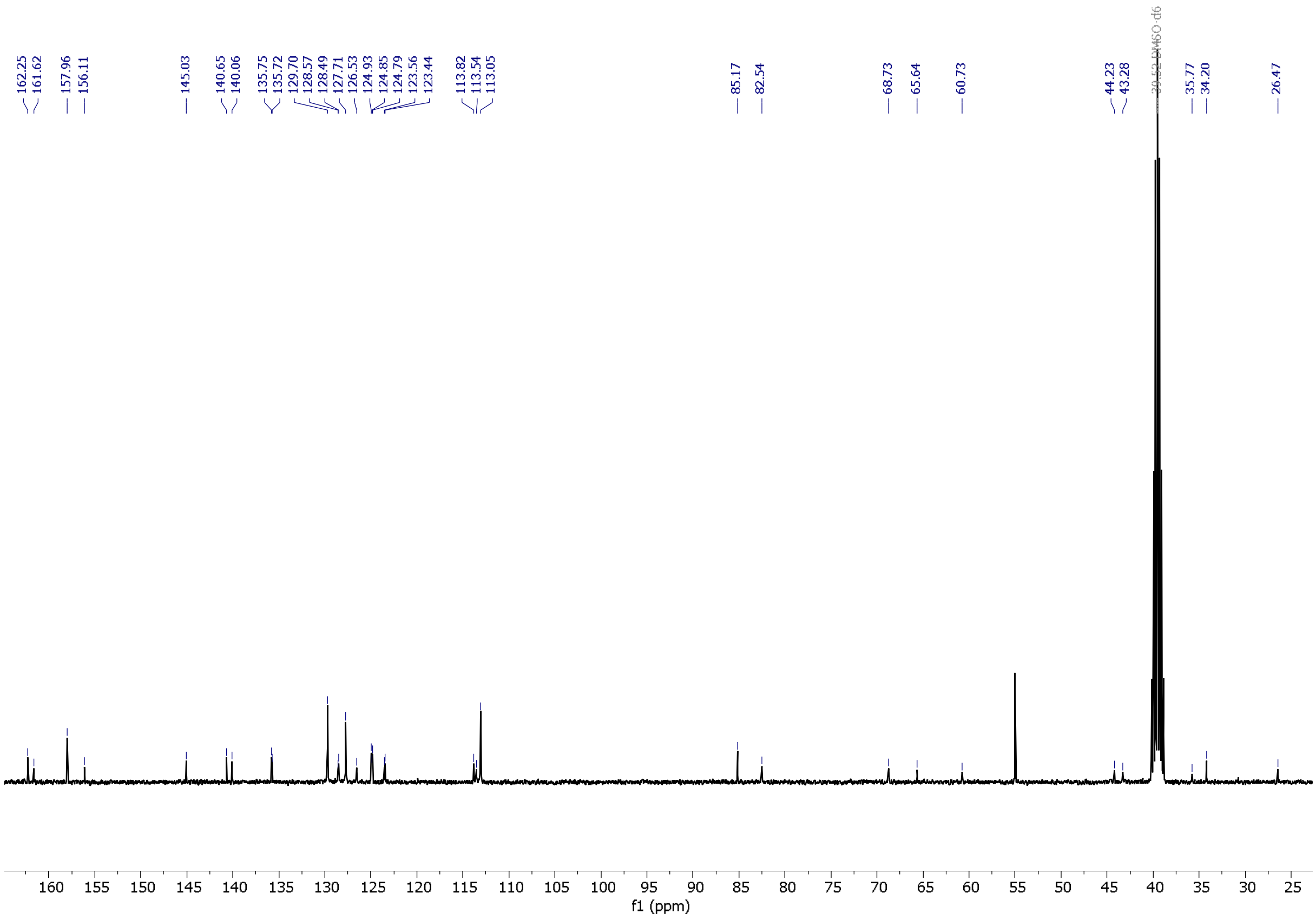

Figure S13: ^{13}C NMR spectrum of **10**.

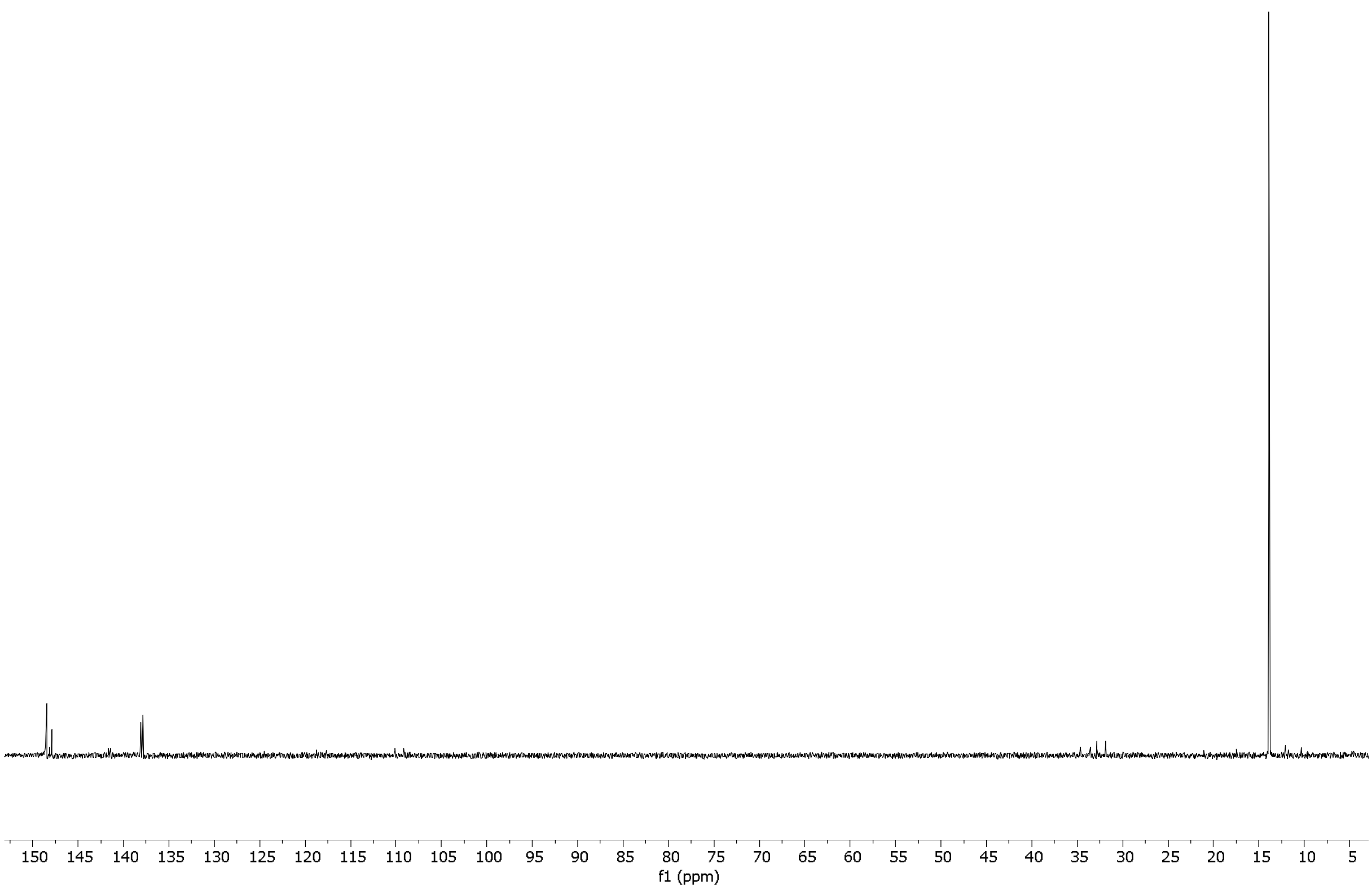

Figure S14: ^{31}P NMR spectrum of **11**.

5. Images of MS analyses

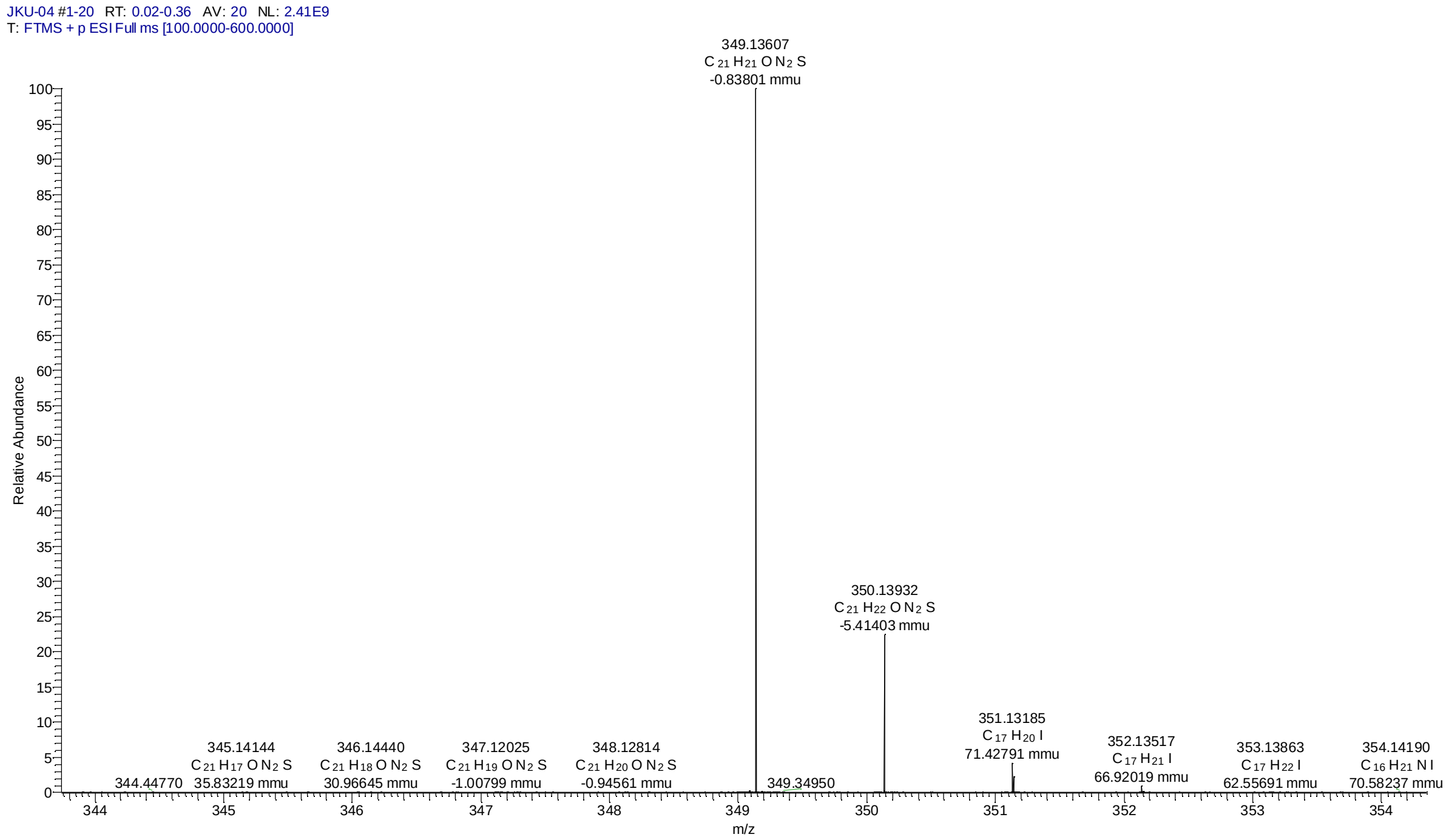

Figure S15: MS analysis of **4**.

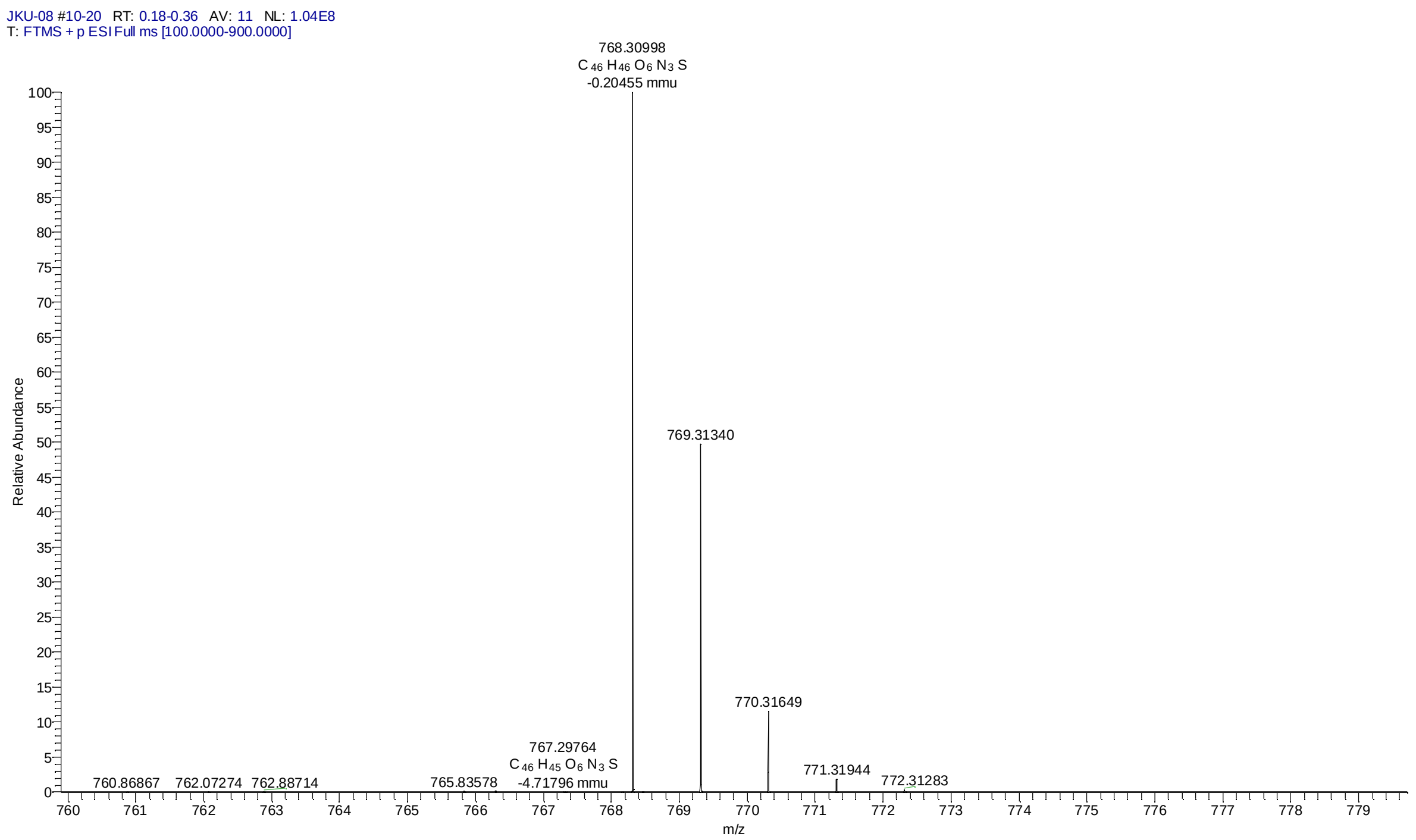

Figure S16: MS analysis of **6**.

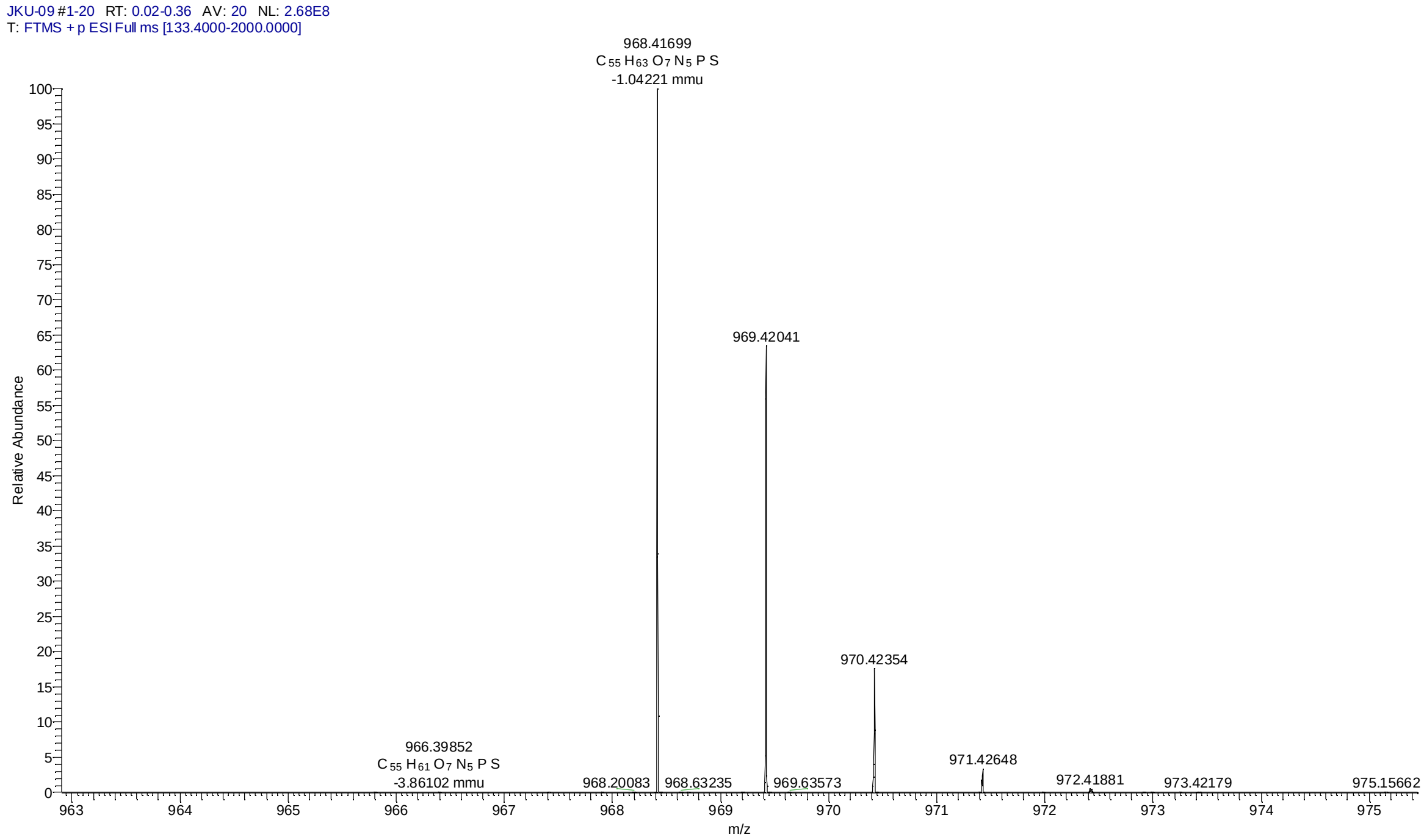

Figure S17: MS analysis of **7**.

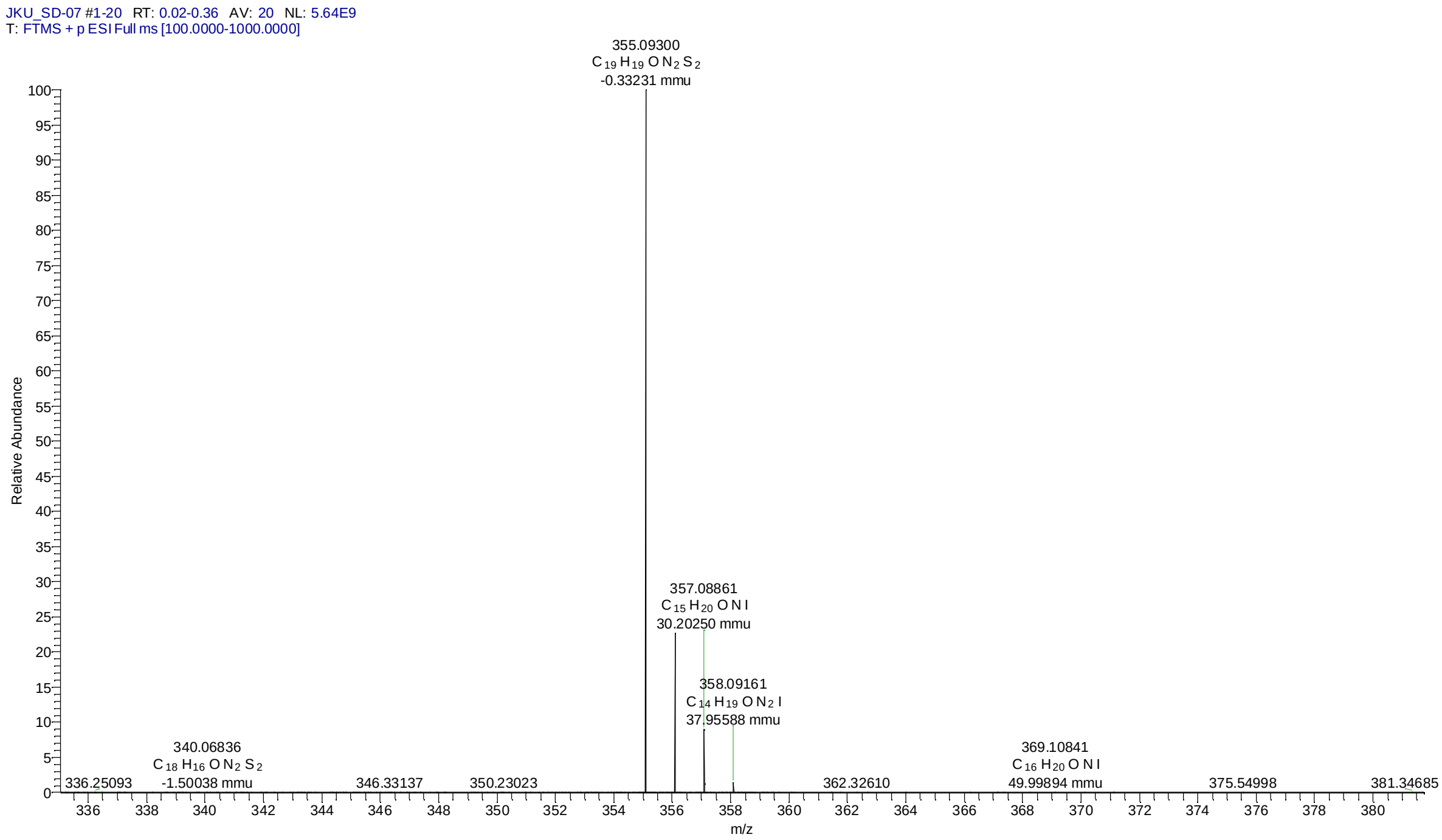

Figure S18: MS analysis of **9**.

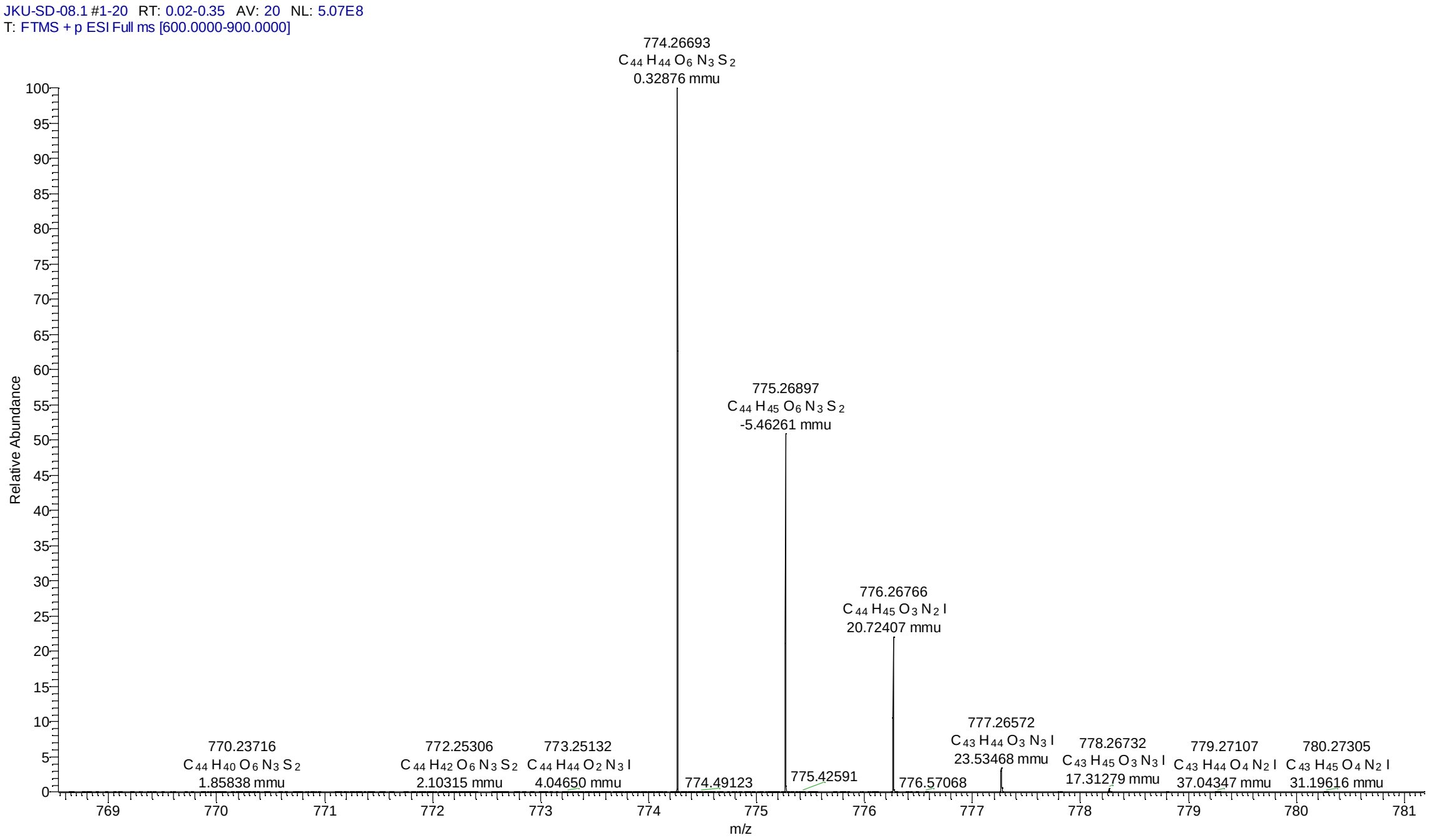

Figure S19: MS analysis of **10**.

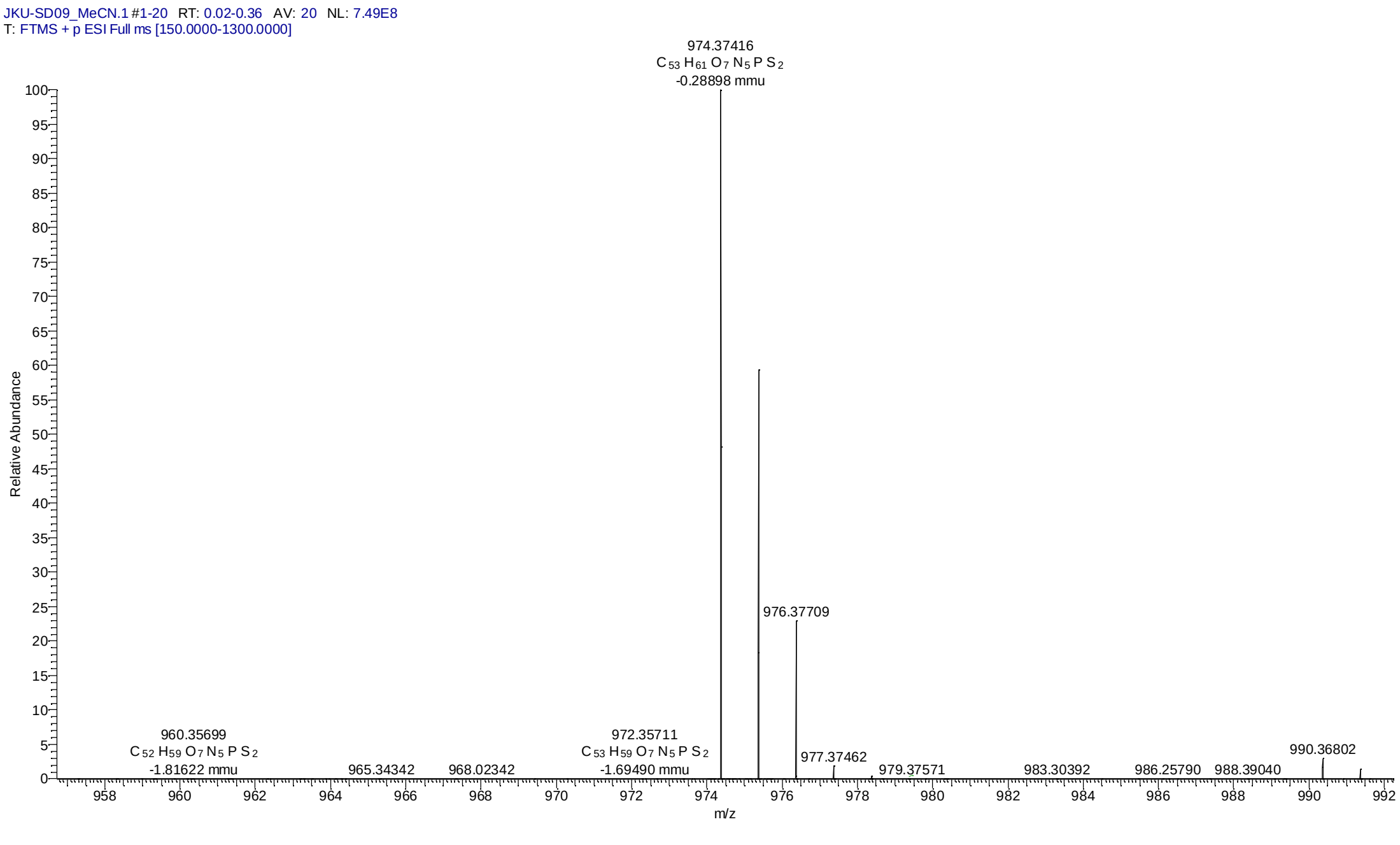

Figure S20: MS analysis of **11**.

6. Synthesis of Oligonucleotides

Table S1: Sequences, calculated and found MALDI-TOF masses of modified oligonucleotides (g/mol).

	Sequence	Mass calculated	Mass found
**TO-DNA1ss**	3´-CAG-TCA-TOTT-GAC-GTA-CG-5´	5407.4	5411.56
**TO-DNA2cs**	5´-GTC-AGT-TTOA-CTG-CAT-GC-3´	5398.4	5403.15
**TO-DNA3ss**	3´-CAG-TCA-TOTO-TTG-ACG-TAC-5´	5606.9	5606.84
**DB-DNA1ss**	3´-CAG-TCA-DBTT-GAC-GTA-CG-5´	5412.9	5416.53
**DB-DNA1cs**	5´-GTC-AGT-TDBA-CTG-CAT-GC-3´	5403.9	5422.46 + Na^+
**DB-DNA3ss**	3´-CAG-TCA-DBDB-TTG-ACG-TAC-5´	5618.0	5642.79 + Na^+

6.1 Images of HPLC analyses

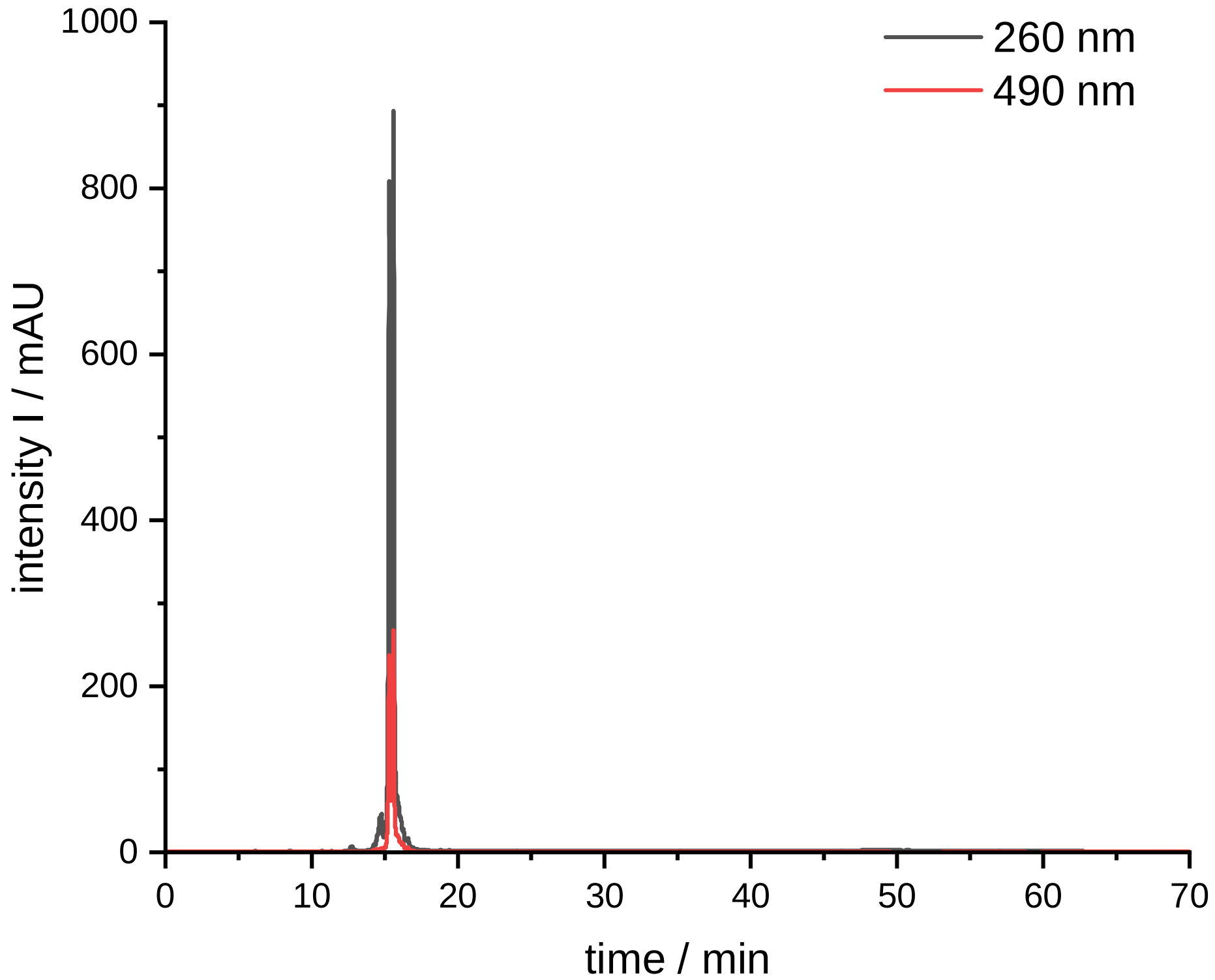

Figure S21: HPLC analysis of **TO-DNA1ss**.

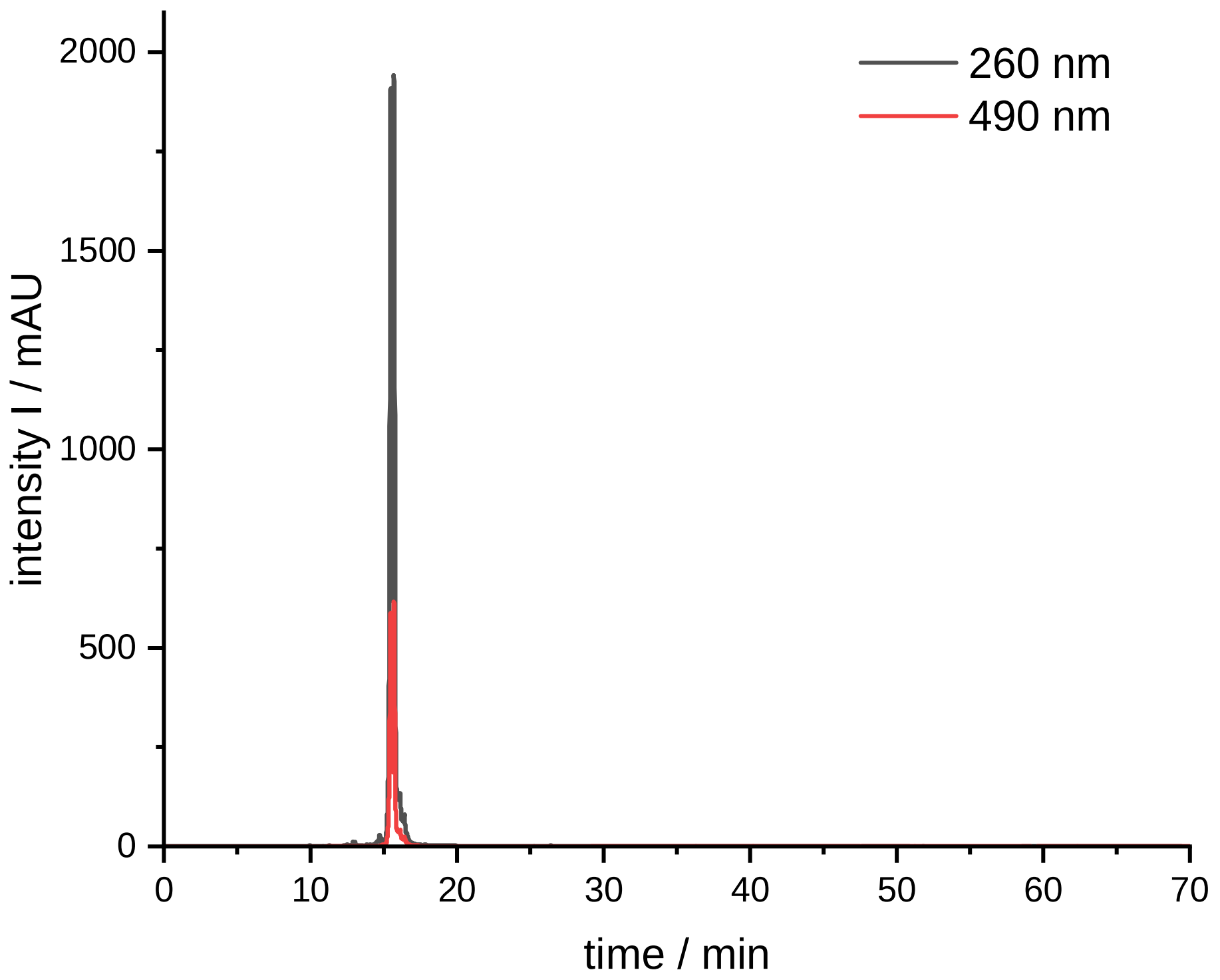

Figure S22: HPLC analysis of **TO-DNA1cs**.

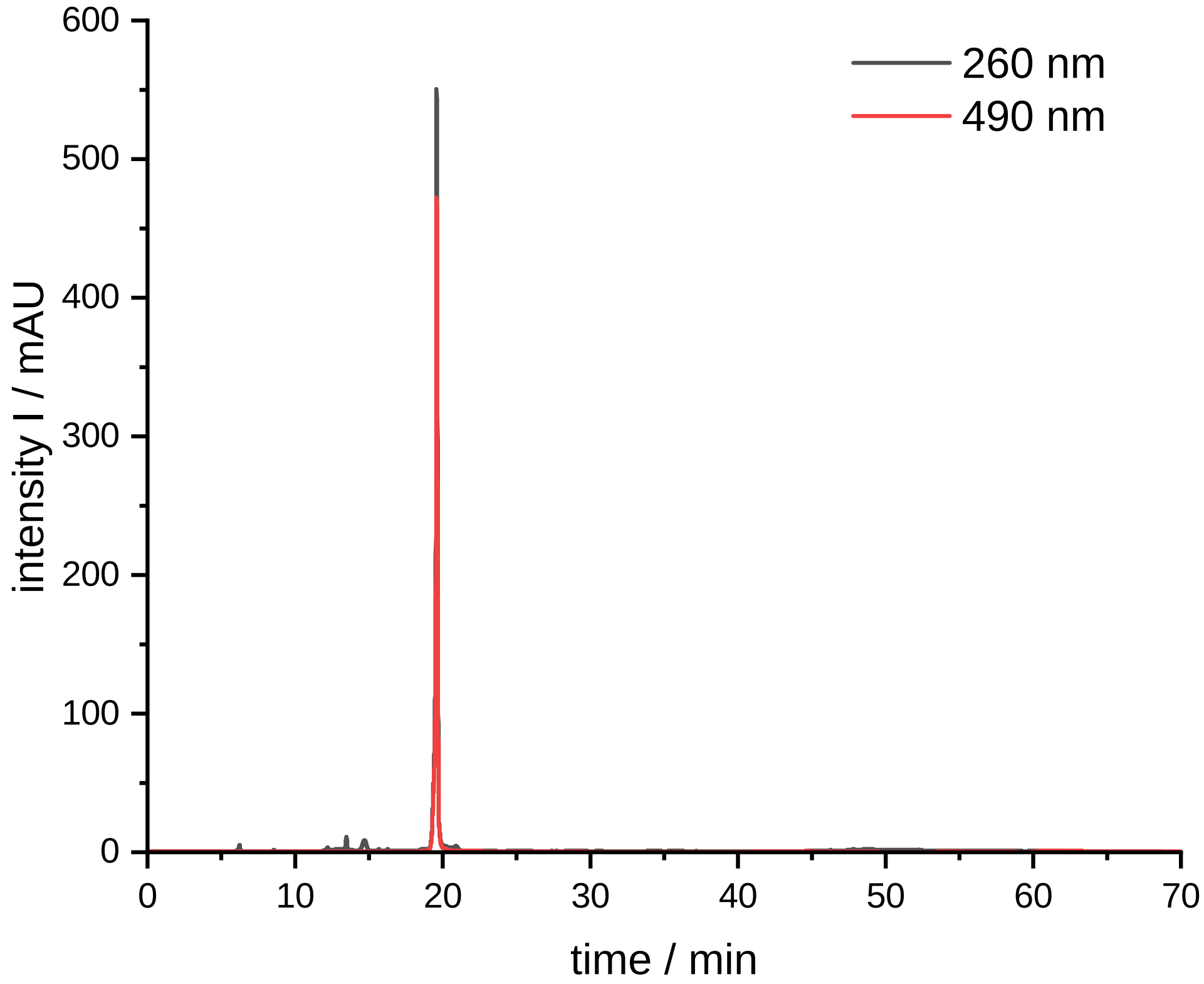

Figure S23: HPLC analysis of **TO-DNA3ss**.

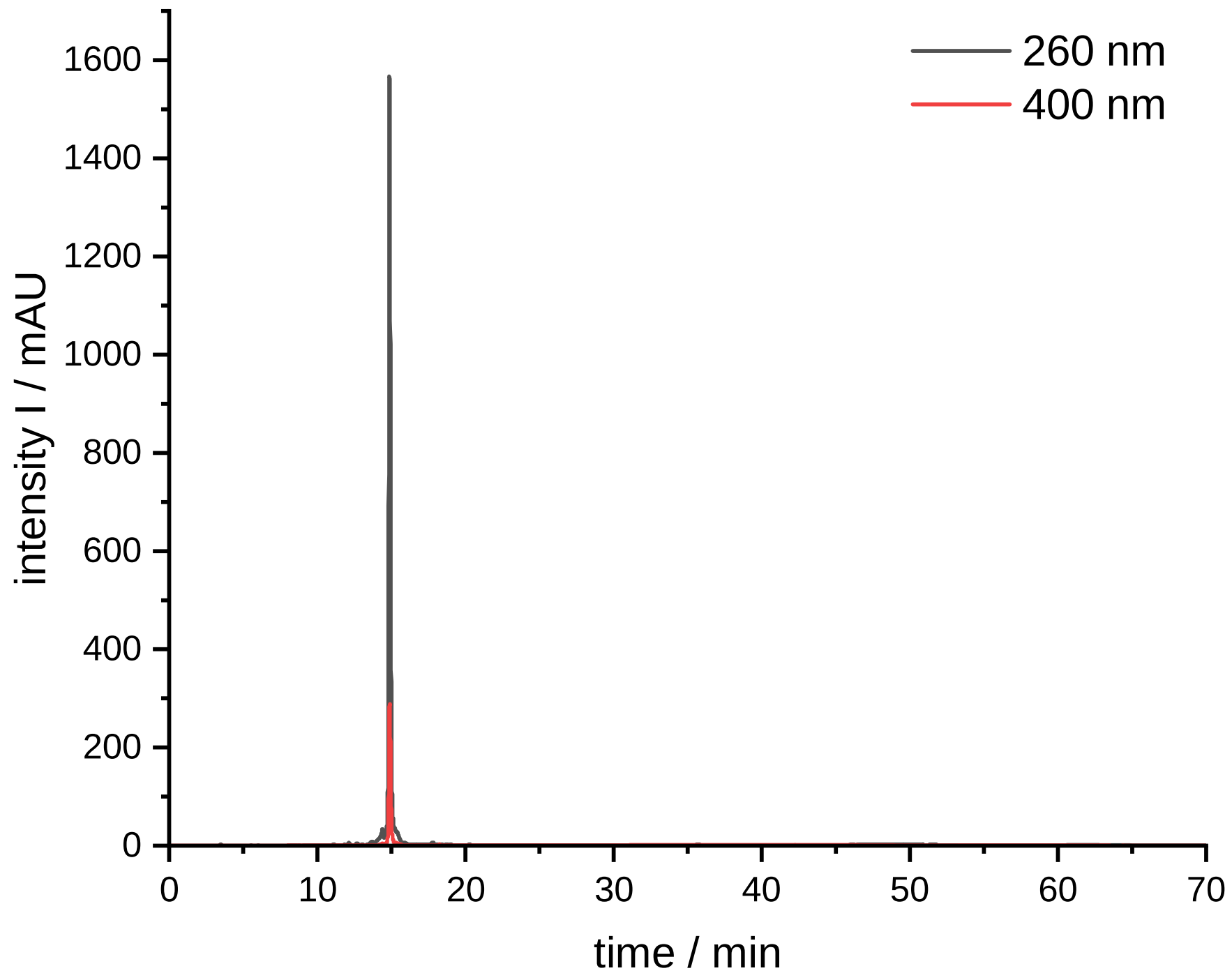

Figure S24: HPLC analysis of **DB-DNA1ss.**

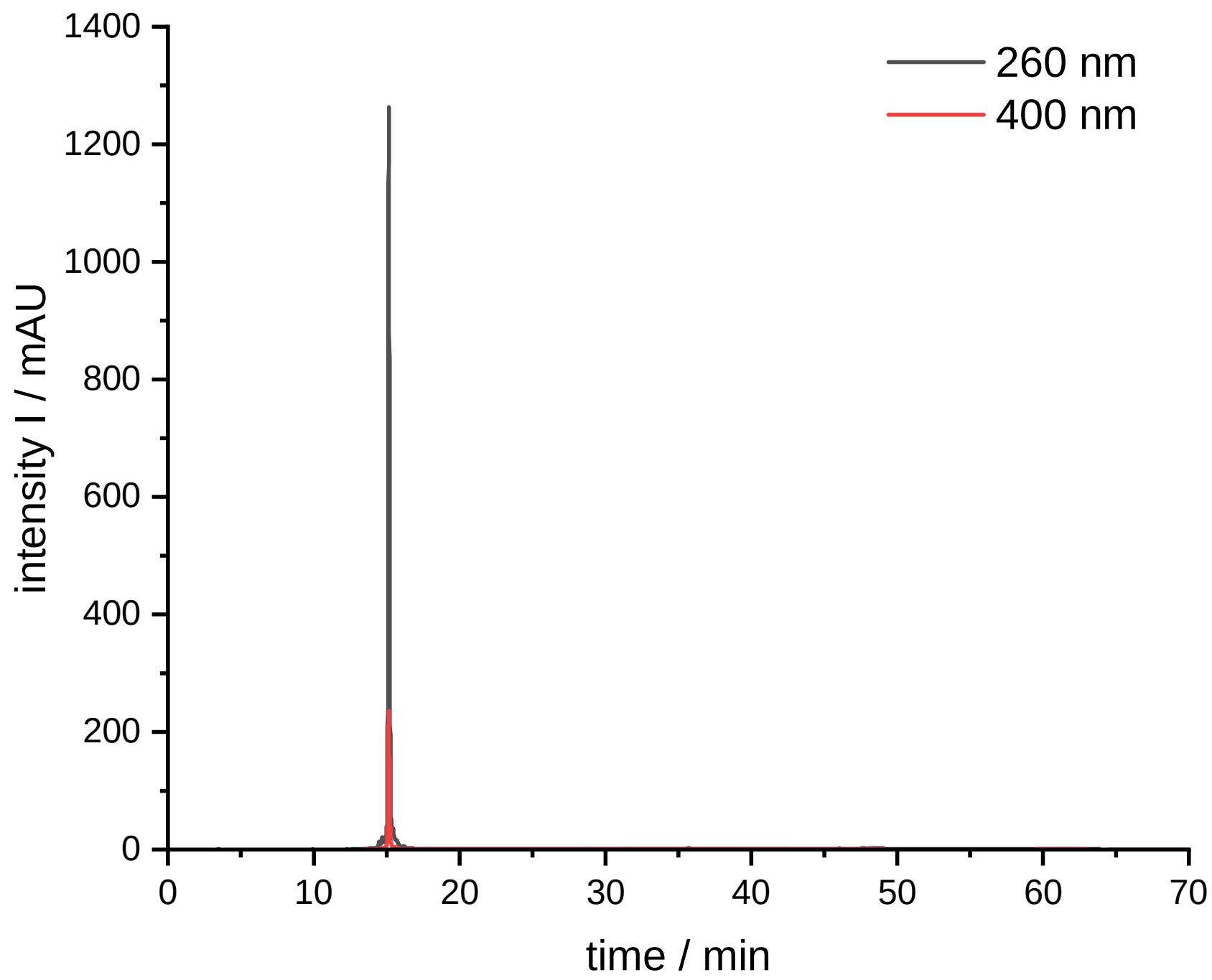

Figure S25: HPLC analysis of **DB-DNA1cs.**

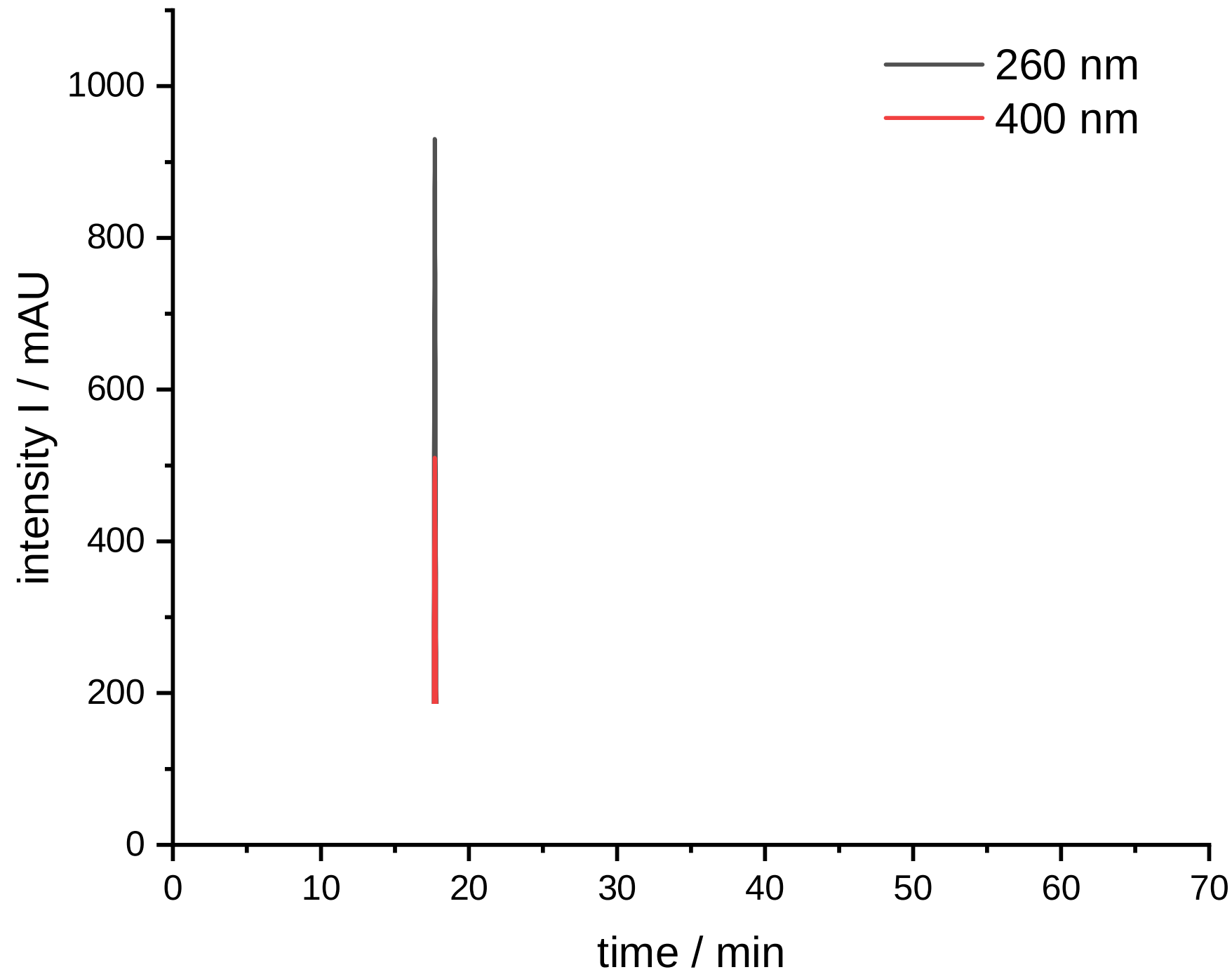

Figure S26: HPLC analysis of **DB-DNA3ss.**

6.2 Images of DNA MS analysis

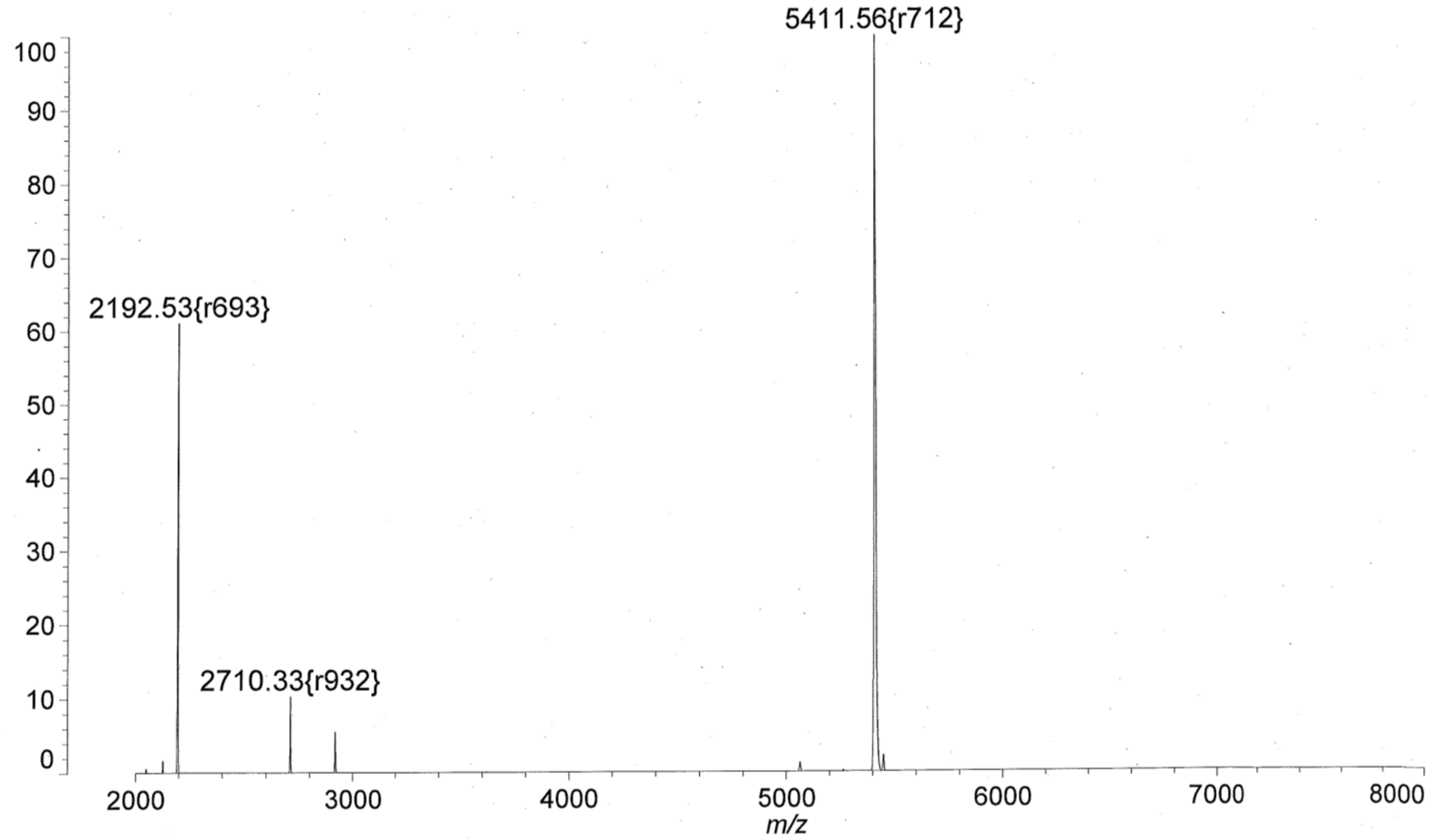

Figure S27: MS analysis of **TO-DNA1ss.**

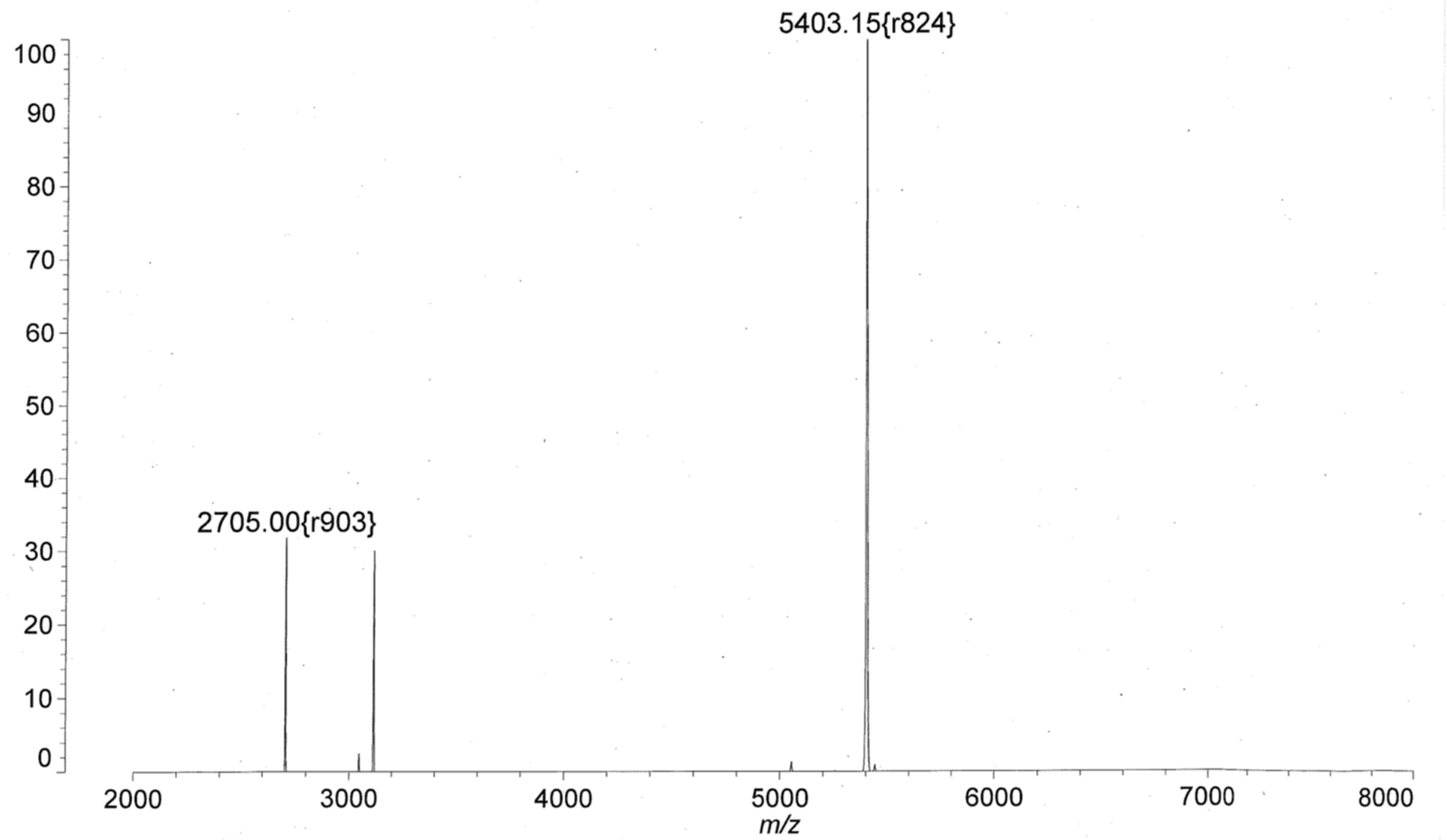

Figure S28: MS analysis of **TO-DNA1cs**.

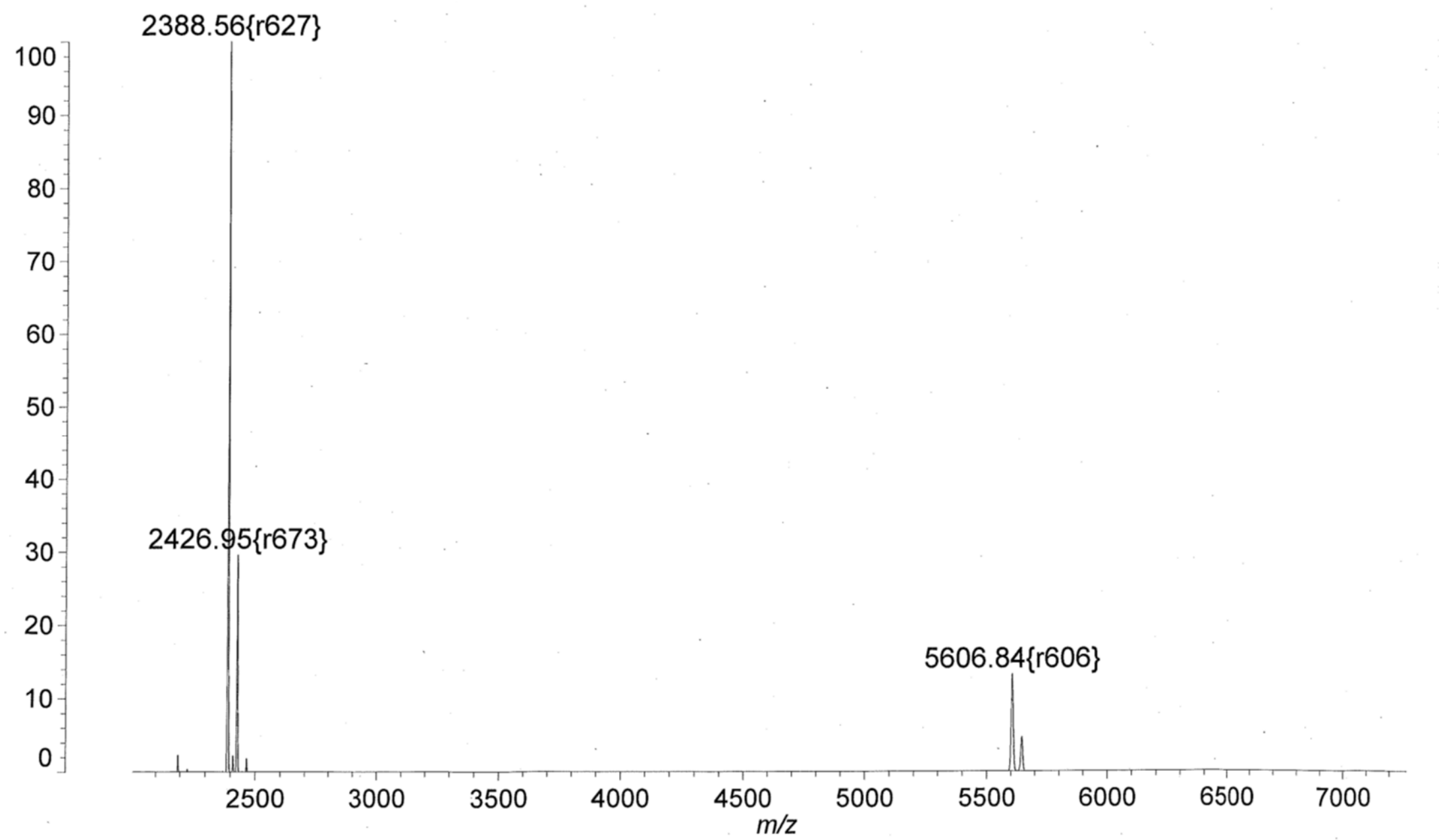

Figure S29: MS analysis of **TO-DNA3ss**.

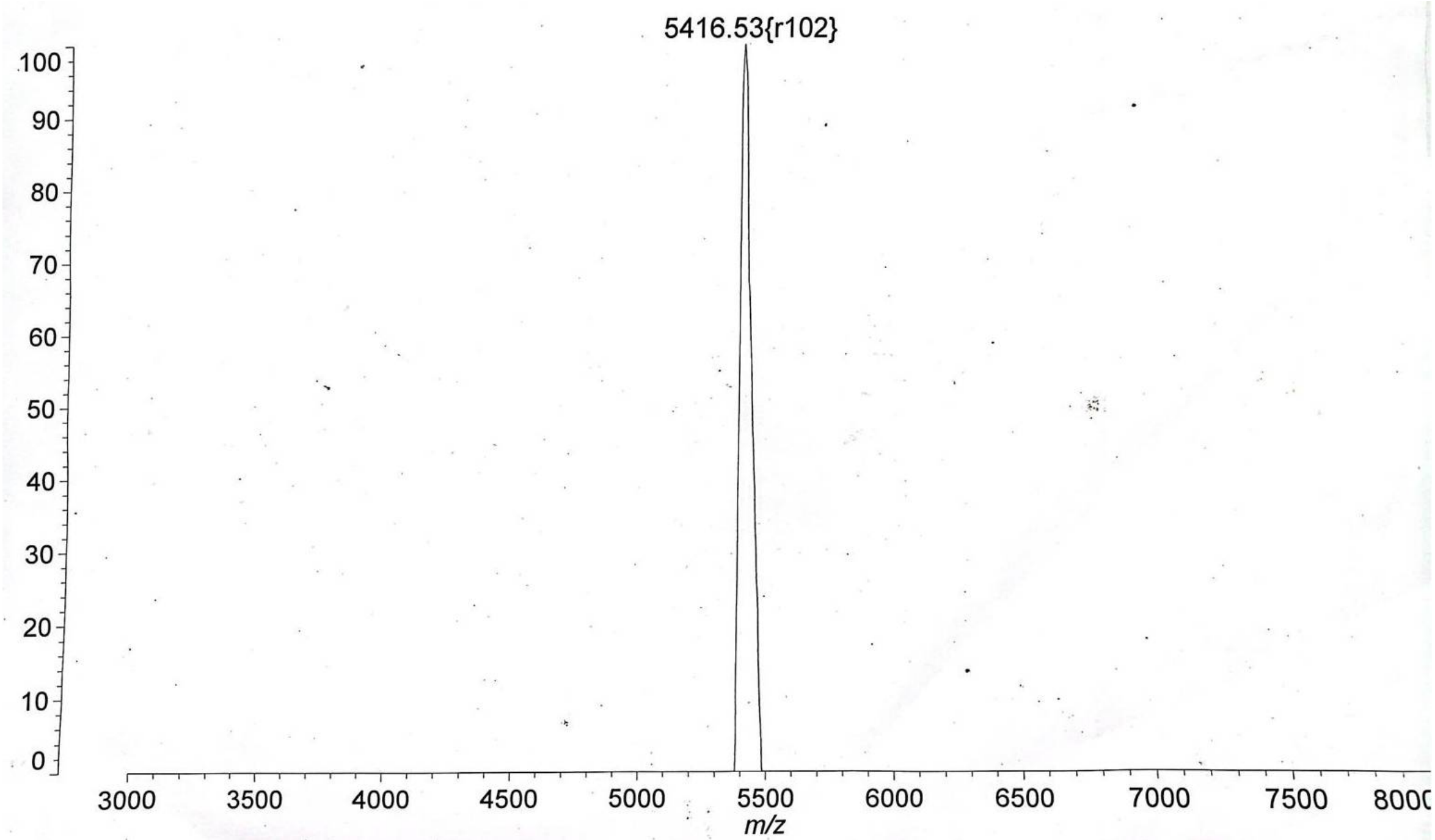

Figure S30: MS analysis **DB-DNA1ss**.

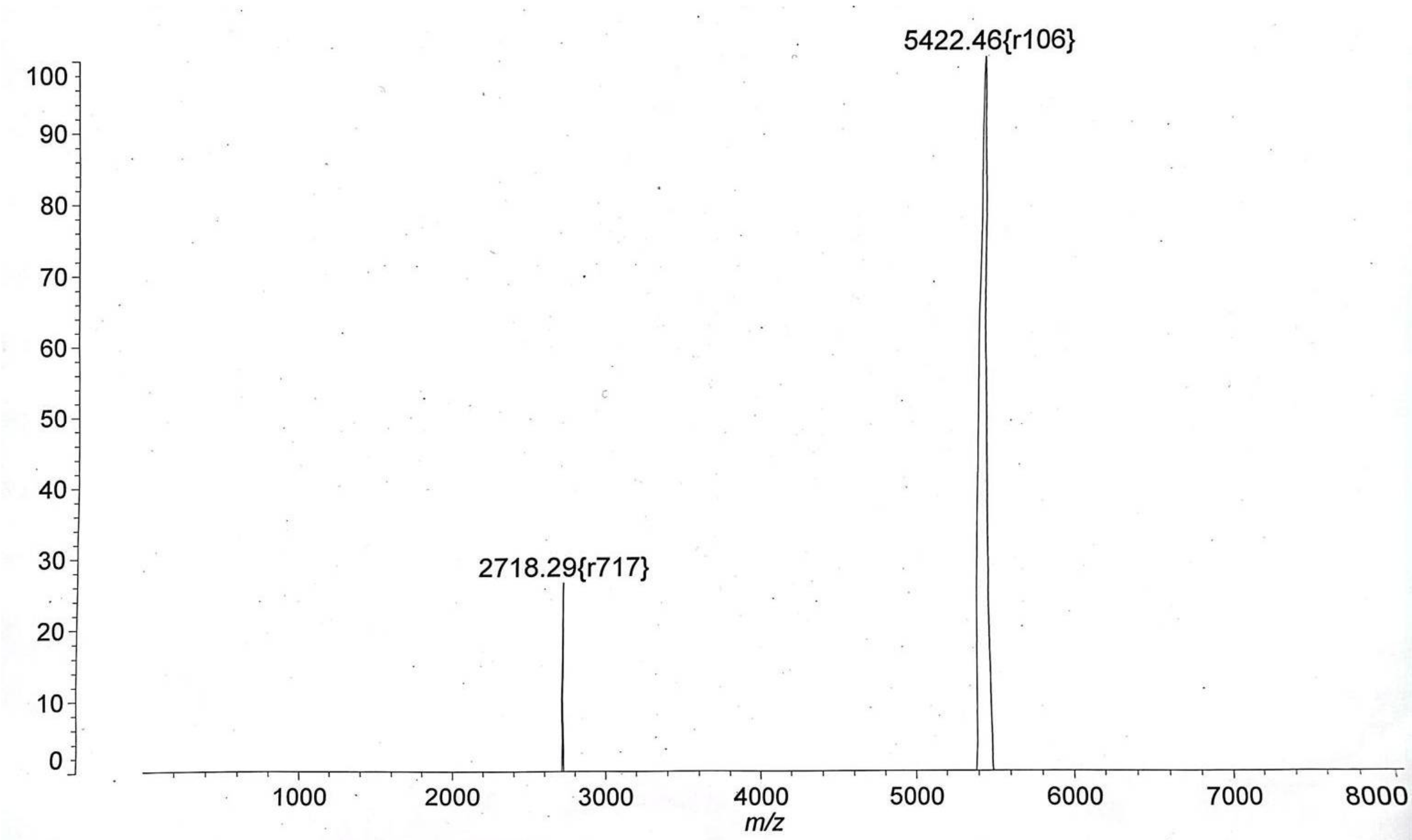

Figure S31: MS analysis of **DB-DNA1cs**.

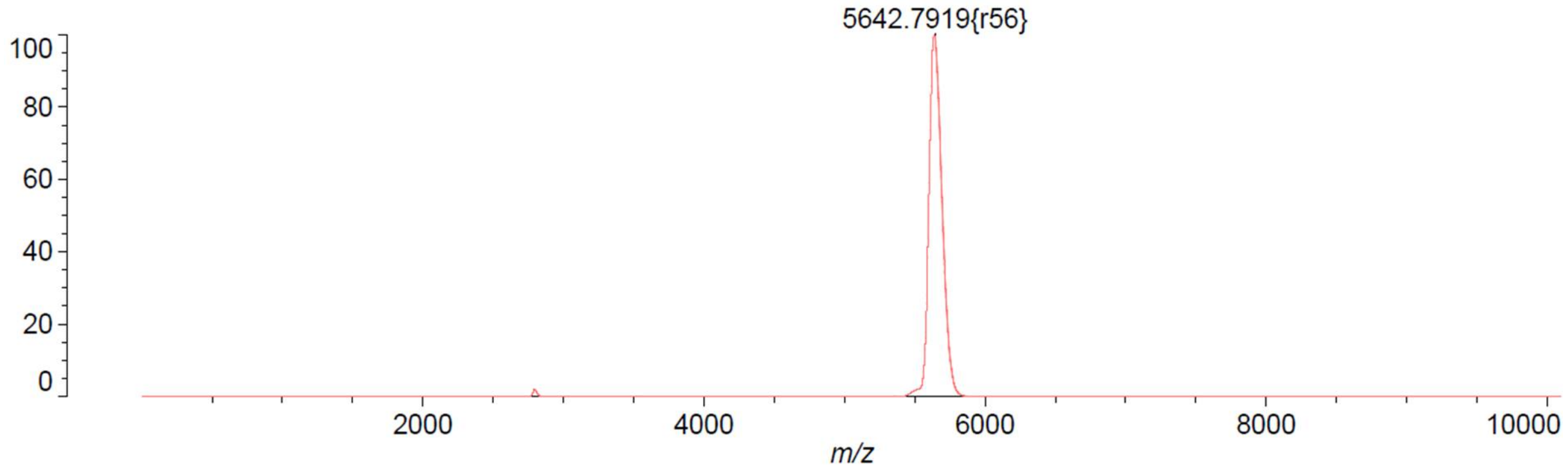

Figure S32: MS analysis of **DB-DNA3ss**.

7. Additional optical spectra of DB-mediated energy transfer

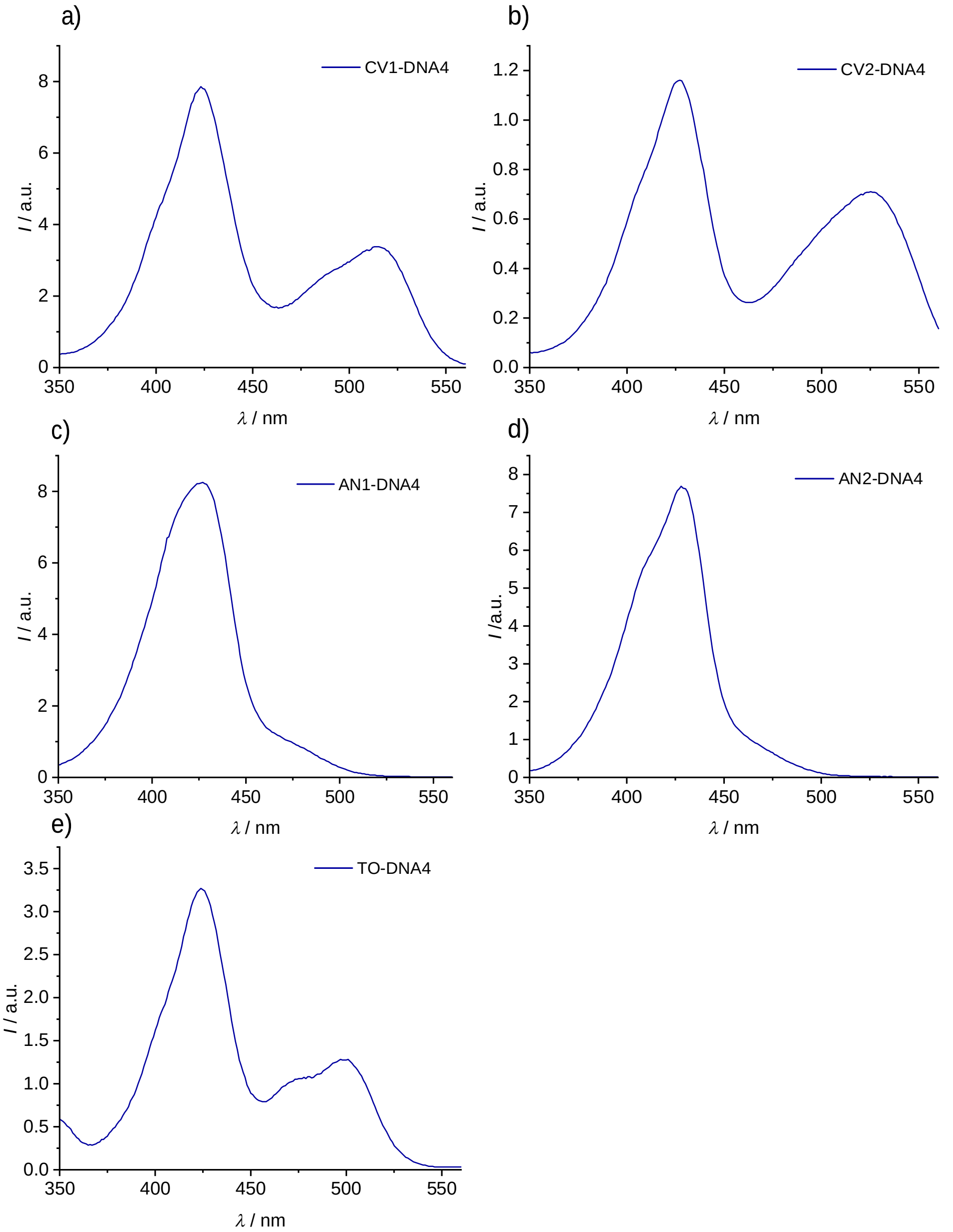

Figure S33: Excitation spectra at λ_{em}=580 nm of a) **CV1-DNA4**; b) **CV2-DNA4**; c) **AN1-DNA4**; d) **AN2-DNA4**; e) **TO-DNA4** (2.5 µM in 10 mM Na-P_i buffer, 250 mM NaCl, pH 7).

8. Synthesis protocol for modified DNA

Table S2: Modified coupling protocol for the single incorporation of **7** (TO) or **11** (DB).

	Branch						
	TIME [0.1 S]	*REAGENT 1*	*REAGENT 2*	*LOCATION*	*S. COL. PTR.*	*COUPLING-TIME [S]*	*BRANCH*
**1**	*4*	*TET*		*COL*	*ON*		
**2**	*6*	*AMD*	*TET*	*COL*			
**3**					*ON*		
**4**						*99*	
**5**						*99*	
**6**						*99*	
**7**						*99*	
**8**						*99*	
**9**	*4*	*AMD*	*TET*	*COL*	*ON*		
**10**					*ON*		
**11**						*99*	
**12**						*99*	
**13**						*99*	
**14**						*99*	
**15**						*99*	
**16**	*6*	*AMD*	*TET*	*COL*	*ON*		
**17**					*ON*		
**18**						*99*	
**19**						*99*	
**20**						*99*	
**21**						*99*	
**22**						*99*	
**23**						*99*	
**24**	*20*	*ACN*		*M_W*			
**25**	*20*	*GAS*		*M_W*			

Table S3: Modified coupling protocol for the double incorporation **7** (TO) or **11** (DB).

	Branch						
	TIME [0.1 S]	*REAGENT 1*	*REAGENT 2*	*LOCATION*	*S. COL. PTR.*	*COUPLING-TIME [S]*	*BRANCH*
**1**	*6*	*TET*		*COL*	*ON*		
**2**	*8*	*AMD*	*TET*	*COL*			
**3**					*ON*		
**4**						*99*	
**5**						*99*	
**6**						*99*	
**7**						*99*	
**8**						*99*	
**9**	*6*	*AMD*	*TET*	*COL*	*ON*		
**10**					*ON*		
**11**						*99*	
**12**						*99*	
**13**						*99*	
**14**						*99*	
**15**						*99*	
**16**	*6*	*AMD*	*TET*	*COL*	*ON*		
**17**					*ON*		
**18**						*99*	
**19**						*99*	
**20**						*99*	
**21**						*99*	
**22**						*99*	
**23**						*99*	
**24**	*20*	*ACN*		*M_W*			
**25**	*20*	*GAS*		*M_W*			
